# Comparative host transcriptome in response to pathogenic fungi identifies common and species-specific transcriptional antifungal host response pathways

**DOI:** 10.1016/j.csbj.2020.12.036

**Published:** 2020-12-26

**Authors:** Mariolina Bruno, Intan M.W. Dewi, Vicky Matzaraki, Rob ter Horst, Marina Pekmezovic, Berenice Rösler, Laszlo Groh, Rutger J. Röring, Vinod Kumar, Yang Li, Agostinho Carvalho, Mihai G. Netea, Jean-Paul Latgé, Mark S. Gresnigt, Frank L. van de Veerdonk

**Affiliations:** a Department of Internal Medicine and Radboudumc Center for Infectious Diseases (RCI), Radboud University Medical Center, Nijmegen, The Netherlands; b Department of Microbial Pathogenicity Mechanisms, Leibniz Institute for Natural Product Research and Infection Biology – Hans Knöll Institute, Beutenbergstraße 11a 07745, Jena, Germany; c Department of Genetics, University of Groningen, University Medical Center Groningen, Groningen, The Netherlands; d Centre for Individualised Infection Medicine (CiiM) and TWINCORE, Joint Ventures between the Helmholtz-Centre for Infection Research (HZI) and the Hannover Medical School (MHH), Hannover, Germany; e Department of Internal Medicine and Radboud Center for Infectious Diseases, Radboud University Medical Center, Nijmegen, The Netherlands; f Life and Health Sciences Research Institute (ICVS), School of Medicine, University of Minho, Braga, Portugal; g ICVS/3B’s – PT Government Associate Laboratory, Guimarães/Braga, Portugal; h Department for Genomics & Immunoregulation, Life and Medical Sciences Institute (LIMES), University of Bonn, 53115 Bonn, Germany; i Unité des Aspergillus, Institut Pasteur, Paris, France; j Junior Research Group Adaptive Pathogenicity Strategies, Leibniz Institute for Natural Product Research and Infection Biology – Hans Knöll Institute, Beutenbergstraße 11a 07745, Jena, Germany

**Keywords:** Host immune response, RNAseq, Transcriptomics of pathogenic fungi, Opportunistic infections, *C. albicans*, *A. fumigatus*, *R. oryzae*, Cytokines, Antifungal core host response, Pattern recognition receptors, Immunometabolism

## Abstract

Candidiasis, aspergillosis, and mucormycosis cause the majority of nosocomial fungal infections in immunocompromised patients. Using an unbiased transcriptional profiling in PBMCs exposed to the fungal species causing these infections, we found a core host response in healthy individuals that may govern effective fungal clearance: it consists of 156 transcripts, involving canonical and non-canonical immune pathways.

Systematic investigation of key steps in antifungal host defense revealed fungal-specific signatures. As previously demonstrated, *Candida albicans* induced type I and Type II interferon-related pathways. In contrast, central pattern recognition receptor, reactive oxygen species production, and host glycolytic pathways were down-regulated in response to *Rhizopus oryzae*, which was associated with an ER-stress response. *TLR5* was identified to be uniquely regulated by *Aspergillus fumigatus* and to control cytokine release in response to this fungus.

In conclusion, our data reveals the transcriptional profiles induced by *C. albicans, A. fumigatus, and R. oryzae*, and describes both the common and specific antifungal host responses that could be exploited for novel therapeutic strategies.

## Graphical abstract

**Figure:**
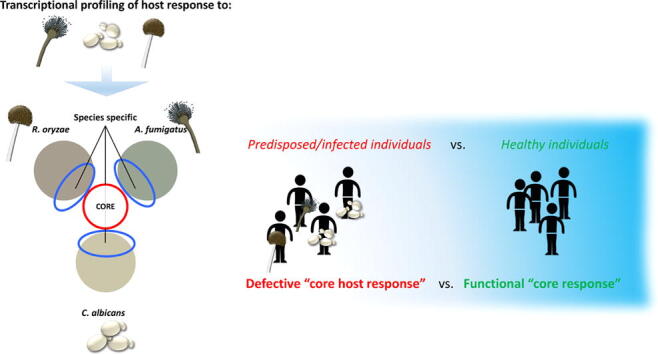

## 1 Introduction

Opportunistic fungal infections are worldwide causes of significant morbidity and mortality that equals either bacterial, viral, or parasitic infections [19], [108]. Among fungal diseases, aspergillosis, candidiasis, and mucormycosis account for the majority of opportunistic infections in immunocompromised patients, with the notable exception of HIV infected patients who primarily suffer from cryptococcosis in addition to oral candidiasis. Infections caused by the three major species *Aspergillus fumigatus, Candida albicans,* and *Rhizopus oryzae,* significantly increased in incidence over the past years from approximately 610.000 to 960.000 life-threatening invasive infections annually, with mortality rates ranging between 30 and 95% collectively [14], [19]. The number of immunocompromised patients is steadily increasing [19], [35] due to evolving medical care with more invasive procedures, increased use of immunosuppressive drugs [26], [75], and growing numbers of ICU admissions. These factors mutually contribute to the increasing incidence of aspergillosis, candidiasis, and mucormycosis.

Although *C. albicans* is a widespread commensal in the human gut [46], [52], it can cause invasive candidiasis when the host immune system is impaired [55], [78], [106], [111] and the microbiome is disrupted, for example, by the use of broad-spectrum antibiotics [11], [117]. In contrast to *C. albicans*, *Aspergillus* species are saprotrophic fungi, which usually do not colonize the human host. However, all humans are exposed to the conidia of these environmental fungi on a daily basis, which can result in severe infections in immunocompromised hosts [57], [86], [107]. Compared to *C. albicans* and *A. fumigatus*, fungi of the genus Mucorales cause invasive disease in a more heterogeneous group of immunocompromised individuals, including diabetes and trauma patients [86]. Mucormycosis is considered one of the most aggressive forms of opportunistic fungal infection [19], [72] and *Rhizopus oryzae* is the most commonly isolated species [85].

The outcome of opportunistic invasive fungal infections remains poor, and therapeutic approaches targeting the immune system have been recently recognized as a potentially lifesaving strategy for patients with invasive fungal infections [6], [103]. To improve antifungal immunotherapy, a global insight into the protective antifungal host response is needed. A common denominator of these three phylogenetically distinct fungal species is that they can all cause infections in immunocompromised patients with similar predisposing factors, including neutropenia, myeloablative and immunosuppressive therapy [15], [38], [51], [60], [64].

We hypothesized that there is a core host response that governs resistance to these three opportunistic pathogens, which is debilitated in immunocompromised patients. Nevertheless, there are also intrinsic differences in the pathogenesis of candidiasis, aspergillosis, and mucormycosis among these patients. Species-specific host defense pathways in terms of pathogen recognition, cytokine release, and metabolism may be activated depending on the particular fungal species encountered by the immune system. The knowledge of these mechanisms will provide valuable insights for the selection of immunotherapies adapted against each species. To characterize the core host response and determine species-specific host responses against *A. fumigatus, C. albicans,* and *R. oryzae*, an unbiased transcriptional profiling approach was used to establish differentially expressed RNA transcripts in human peripheral blood mononuclear cells (PBMCs) in response to these 3 fungal species. Subsequently, the most relevant pathways core and species-specific pathways have been functionally validated.

## 2 Results

### 2.1 Transcriptional profiling of host response to *A. fumigatus, C. albicans*, and *R. oryzae*

**Fig. 1:**
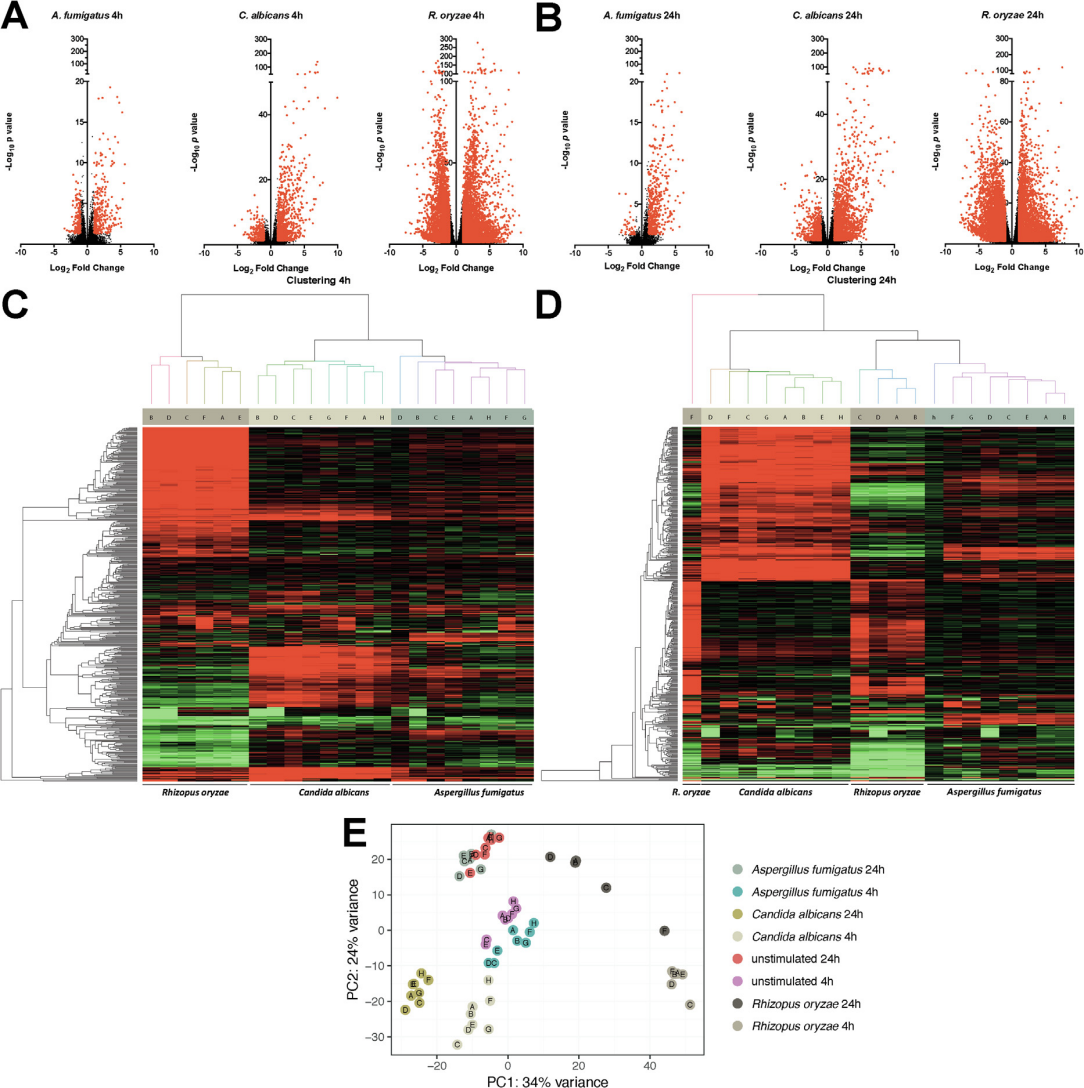
Transcriptional response to *A. fumigatus, C. albicans,* and *R. oryzae* (A-B) Volcano plots of differentially regulated genes assessed by RNA-Seq in human PBMCs from healthy volunteers in response stimulation with *A. fumigatus* (n = 8 donors)*, C. albicans* (n = 8 donors), or *R. oryzae* (n = 6 and 5 donors for 4 and 24 h respectively) for 4 h (A) or 24 h (B). The plots represent Log_2_ fold change and -Log_10_ of the corrected *p*-value compared to the unstimulated control. Transcripts with a corrected *p*-value < 0.05 and a mean Log_2_FC > 1 of < −1 are plotted in red and were considered as differentially regulated. (D-E) Heatmap showing unsupervised clustering analysis of the transcriptional responses of PBMCs to the three fungal species. Distances were defined as 1 - Pearson correlation and average-linkage clustering was used. Numbers 1–8 on the branches of the dendrogram represent numbers of the respective PBMC donors after 4 h (C) and 24 h (D) of stimulation. After QC 2 donors stimulated for 4 h and three donors stimulated for 24 h with *R. oryzae* needed to be excluded. (E) Principal component analysis of the transcriptional response signatures. Data from each individual sample is plotted along the two main principal components (PC1, PC2). The two principal components account for 24–34% of the total variance in gene expression (PC1 34%; PC2 24%). Samples from the various stimulations are color coded as follows: *C. albicans* 4 h (light beige) and 24 h (golden), *A. fumigatus* 4 h (teal blue) and 24 h (blue grey), *R. oryzae* 4 h (dark beige) and 24 h (brown), and unstimulated 4 h (Purple) and 24 h (light red). (For interpretation of the references to color in this figure legend, the reader is referred to the web version of this article.)

The genome-wide transcriptional response of human PBMCs in response to *A. fumigatus, C. albicans,* and *R. oryzae* was assessed using RNA sequencing (RNA-Seq). Unstimulated PBMCs served as reference, and the transcriptional profile was assessed after 4 h (4 h) and 24 h (24 h) of stimulation with inactivated yeast and conidia of the three fungal species. Transcripts that exhibited a Log_2_fold change >1 or <−1 compared to unstimulated PBMCs and an adjusted *p-*value of < 0.05 were considered differentially regulated. *A. fumigatus* differentially regulated 472 transcripts (290 up and 182 down) at 4 h, and 506 transcripts (467 up and 39 down) at 24 h (Fig. 1A, B). *C. albicans* differentially regulated 1055 transcripts (821 up and 234 down) at 4 h, and 2448 transcripts (1733 up and 715 down) at 24 h (Fig. 1A, B). *R. oryzae* differentially regulated a staggering amount of 10,104 transcripts (6043 up and 4061 down) at 4 h, and 9646 transcripts (4974 up and 4672 down) at 24 h (Fig. 1A, B). Unsupervised hierarchical clustering revealed that the transcriptional response of PBMCs from various donors show stimulus dependent clustering at both the 4 h and 24 h time points (Fig. 1C, D). Of note, the response induced by *R. oryzae* of a single donor at 24 h was observed to form a separate cluster (Fig. 1D). Following the observation that different expression profiles were induced by the three fungi in the unsupervised hierarchical cluster analysis, the inter-fungal heterogeneity in the response of PBMCs was investigated. Principle component analysis (PCA) revealed that the majority of the variance in transcription was stimulus-dependent, as demonstrated by the three distinct clusters corresponding to the three different fungal species. Of note, the response to *A. fumigatus* stimulation clustered closely to the unstimulated cells and the response to *R. oryzae* showed high inter-individual variation (Fig. 1E).

### 2.2 Common transcriptional response to *A. fumigatus, C. albicans,* and *R. oryzae*

**Fig. 2:**
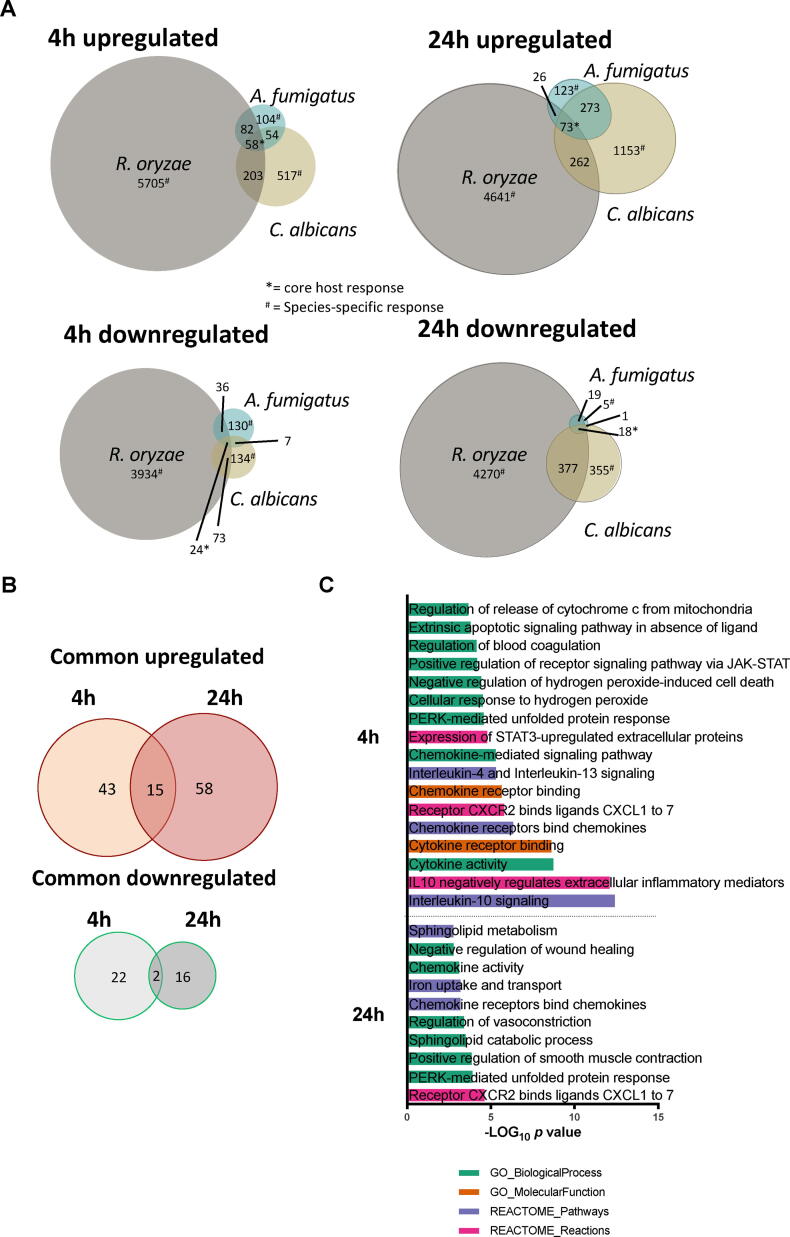
Common transcriptional response to *A. fumigatus, C. albicans* and *R. oryzae* (A) Proportional VENN diagrams of differentially regulated RNA transcripts from the RNA-Seq dataset (corrected *p*-values < 0.05 and a Log_2_FC > 0.9 for up-regulated and < -0.9 for down-regulated; see methods) and their overlap in PBMCs stimulated with *A. fumigatus, C. albicans* or *R. oryzae* after 4 h (left) or 24 h (right). (B) Proportional VENN diagram representing the temporal distribution of RNA transcripts that are commonly regulated in PBMCs following stimulation by all of the three fungi *A. fumigatus, C. albicans* or *R. oryzae* and represent the “core” host response. (C) Enriched pathways within the set of commonly differentially regulated genes plotted as the -Log_10_ of the *p*-value after Benjamini-Hochberg correction.

Subsequently, the common transcriptional responses to the three different fungal species were investigated at both time-points (Fig. 2A). Despite major differences in the number of differentially regulated genes (Fig. 1A-C)*,* 82 genes (58 up-regulated and 24 down-regulated) were commonly regulated by all three fungal species after 4 h of stimulation, and 91 (73 up-regulated and 18 down-regulated) after 24 h of stimulation (Fig. 2A* and B). Genes involved in host defense such as cytokine and chemokine signaling, phagosome maturation, as well as coagulation, and lncRNAs were overrepresented at both 4 h and 24 h. Pathway enrichment analysis using the commonly up-regulated protein-coding genes confirmed these findings (Fig. 2C). A significant enrichment of *interleukin-10 signaling* (*p* = 4.01 × 10^−13^ REACTOME), *Chemokine receptors bind chemokines* (*p* = 4.68 × 10^-7^ REACTOME), *cytokine receptor binding* (*p* = 2.43 × 10^-9^ GO Molecular Function), and *Regulation of blood coagulation* (*p* = 7.19 × 10^-5^ GO Biological Process) was observed at 4 h. Enriched up-regulated pathways at 24 h included *Receptor CXCR2 binds ligands CXCL1 to 7* (*p* = 2.39 × 10^-5^ REACTOME), *Iron uptake and transport* (*p* = 6.68 × 10^-4^ REACTOME), *sphingolipid catabolic process* (*p* = 3.22 × 10^-4^ GO biological process) and *PERK-mediated unfolded protein response* (*p* = 1.27 × 10^-4^ GO biological process). Key coagulation genes (*F3, PLEK, PLAU,* and *PLAUR*) were up-regulated after 4 h of stimulation with all the three fungi, posing the coagulation cascade as an early core host response. Previous studies have demonstrated associations between coagulation and antifungal host defiance [31], [37], [102]. When exaggerated, this “protective” host response can become detrimental, by causing fungal sepsis-induced coagulopathy, which can be thrombotic or hemorrhagic [56], [74], [82]. As the coagulation pathway is identified as a common response to phylogenetically diverse fungi, it provides a rationale for diagnostic assessment of coagulation in patients with invasive fungal infection, which may aid the allocation of thromboembolic protective therapeutic measures.

No significant enriched pathways were observed in the common down-regulated genes. Only 2 genes, namely *CCR2* and *CD101*, were down-regulated both after 4 h and 24 h stimulation with all the three fungal species (Fig. 2B). Interestingly, using a whole blood infection model a recent study showed only a common up-regulation of genes in response to four tested *Candida* species, while no common gene down-regulation was observed [49]. An overview of the common upregulated and downregulated genes can be found in the related Data in Brief article [20].

### 2.3 Functional validation of the core host response to *A. fumigatus, C. albicans*, and *R. oryzae*

**Fig. 3:**
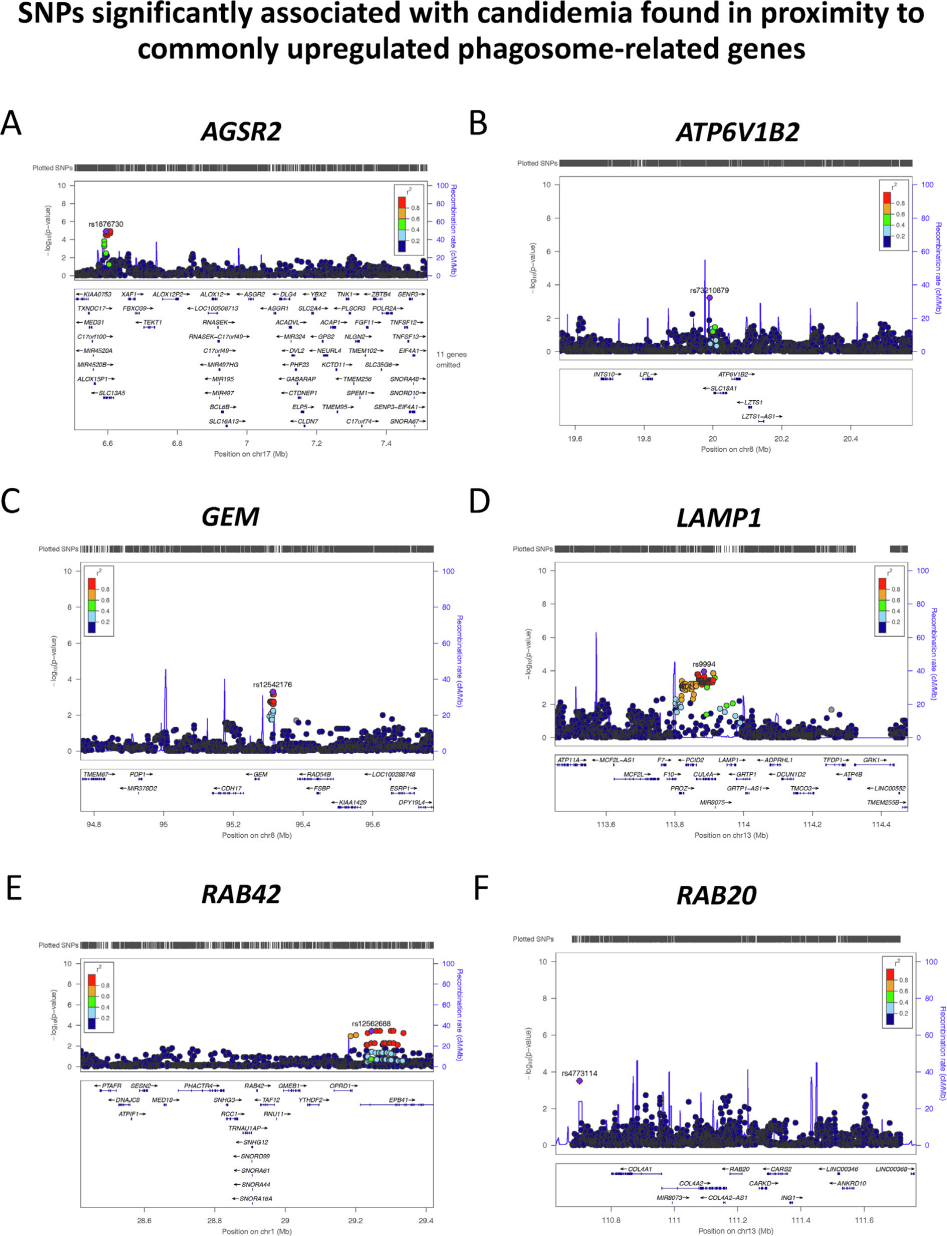
Differentially expressed genes in response to the three fungi are found to be located in candidemia-associated loci (*p* < 0.0006); related to Table 1. Genetic association study in candidemia patient to validate the importance of phagocytosis-associated genes that were differentially regulated in PBMCs of healthy volunteers. (A) Regional association plot of candidemia-associated rs1876730, in close proximity to *AGSR2* gene on chromosome 17. (B) Regional association plot for candidemia-associated SNP rs73210879, which was mapped in a window of 1 MB around *ATP6V1B2* gene on chromosome 8. (C) Regional association plot for candidemia-associated SNP rs12542176, in close proximity to GEM gene on chromosome 8. (D) Regional association plot for candidemia-associated SNP rs9994, in close proximity to *LAMP1* gene on chromosome 13. (E) Regional association plot for candidemia-associated SNP rs12562688, in close proximity to *RAB42* gene on chromosome 1. SNPs are plotted as the -Log of the *p*-value. (F) Regional association plot for candidemia-associated SNP rs4773114, which was mapped in a window of 1 MB around *RAB20* gene on chromosome 13. Local linkage disequilibrium (LD) structure is reflected by the plotted estimated recombination rates (from HapMap) in the region around the associated SNP (purple diamond) and its correlation proxies. The correlation of the lead SNP to other SNPs at the locus is indicated by color. (For interpretation of the references to color in this figure legend, the reader is referred to the web version of this article.)

Despite not enriched as a pathway *per se*, numerous genes involved in phagocytosis, phagosome maturation, endocytosis, and actin remodeling were up-regulated in response to all three fungi. Plasma membrane phosphoinositides [109] and RAB GTPases [114] mediate engulfment and vesicle fusion dynamics with the phagosome to form the mature phagolysosome. Interestingly, *RAB42* and *RAB20*, known to be involved in vesicular remodeling [114], were commonly up-regulated by the three fungi. To validate the involvement of these differentially regulated phagocytosis genes in antifungal host defense; genetic variation in the loci was associated with the risk of invasive candidiasis. To this end, a previously published genome wide association study (GWAS) on candidemia susceptibility [70] was used to test whether genetic variants in a 1 MB window around the genes of interest were associated to candidemia susceptibility. Six intronic and intergenic genetic variants demonstrated significant association (*p* < 5 × 10^-4^) in proximity to the genes: *ASGR2, LAMP1, RAB20, RAB42, GEM*, and *ATP6V1B2* (Table 1). The minor allele of rs4773114 increases susceptibility to candidemia (OR > 1), and the rest of the SNPs are protective against the disease (OR < 1) (Fig. 3A-F, Table 1). Genetic variants within non-coding regions can regulate genes through different mechanisms. Intronic variants can affect mRNA splicing or could also be relevant as enhancers that could regulate expression [27], also known as expression quantitative trait loci (eQTL). To address this point, the *QTLbase* repository [119] has been consulted: the eQTL query from publicly available databases of the six discovered candidemia-associated SNPs have shown that rs4773114 is a known eQTL for *RAB20* gene expression in blood (*p* = 0.031) and rs9994 influences the gene expression of *LAMP1* in monocytes after LPS and IFNα exposure (*p* < 0.0001) (Supplementary Table 1).

**Table 1:** SNPs associated with candidemia susceptibility (*p* < 0.0006) in a window of 1 MB (500 kb around each gene).

SNP	chr	pos	Gene in proximity to SNP	A1	F_A	F_U	A2	P.value	OR
rs1876730	17	6,594,869	ASGR2	C	0.03416	0.1283	T	1.24E-05	0.2403
rs9994	13	113,884,301	LAMP1	C	0.1894	0.3257	T	0.000112	0.484
rs4773114	13	110,699,971	RAB20	T	0.5466	0.4013	G	0.0003123	1.798
rs12562688	1	29,244,944	RAB42	G	0.03727	0.1118	A	0.0003608	0.3074
rs12542176	8	95,312,910	GEM	C	0.1242	0.2303	G	0.0005167	0.4742
rs73210879	8	19,990,150	ATP6V1B2	A	0.1894	0.3092	C	0.000594	0.5221

Legend: OR: odds ratio; A1: minor allele; F_A: Frequency of this allele in cases; F_U: Frequency of this allele in controls; A2: Major allele.

**Fig. 4:**
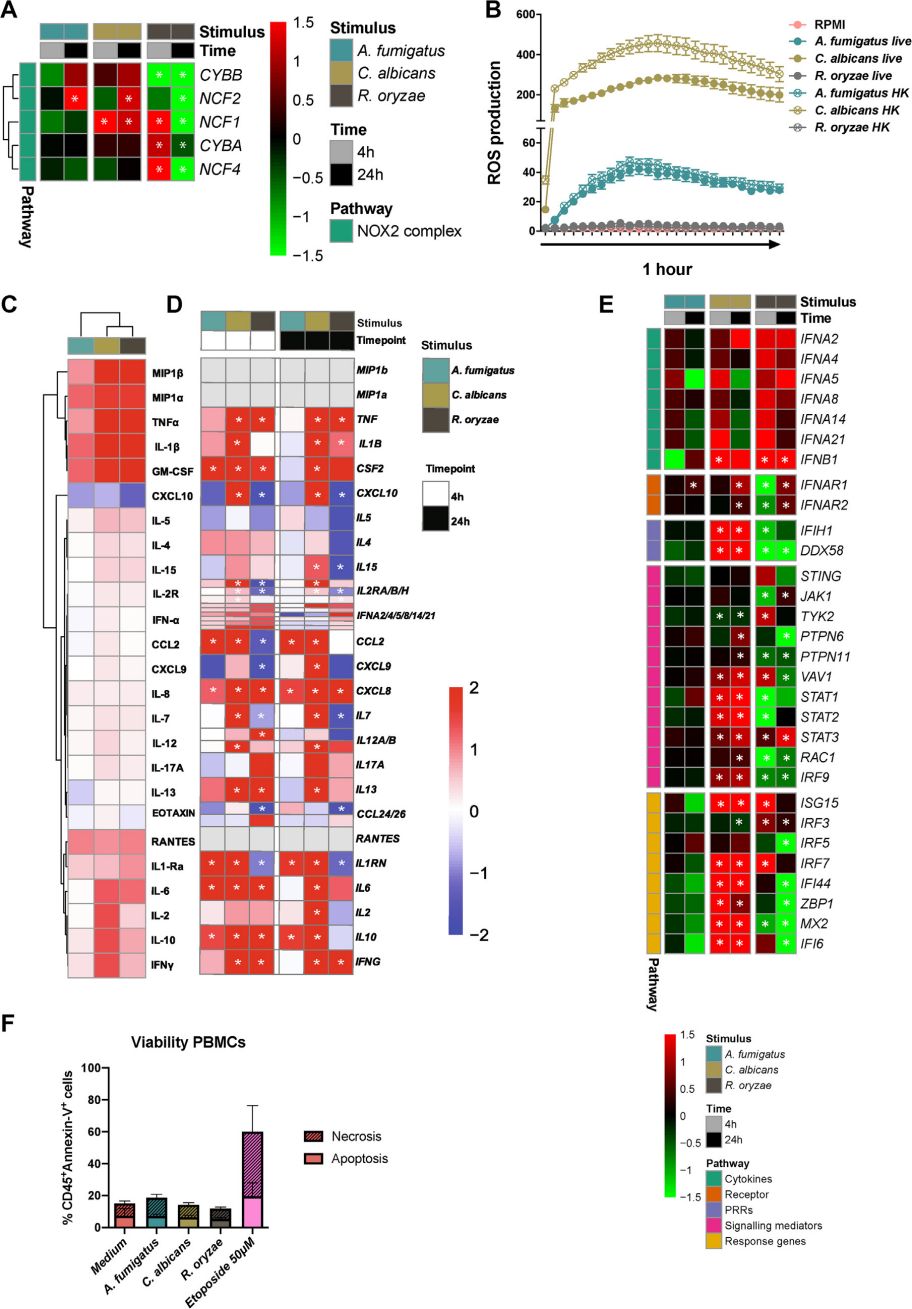
Comparative host response profile of human PBMCs (A) Heatmap of transcriptional expression of NADPH-oxidase 2 subunits extracted from the RNA-Seq dataset of PBMCs stimulated with *A. fumigatus*, *C. albicans* and *R. oryzae* for 4 h and 24 h compared to unstimulated cells (RPMI). The color indicates the Log_2_FC (Shades of red indicate upregulation, while shades of green downregulation, see legend; the white asterisk indicates genes with an adjusted *p*-value < 0.05). (B) Time-course during one hour of PBMCs from healthy volunteers stimulated with live and heat-killed forms of *A. fumigatus*, *C. albicans* and *R. oryzae.* Measurements (ROS induction, RLU/sec) were taken within 1 h in intervals of 2.23 min (n = 6 donors). (C) Heatmaps of cytokine production measured in culture supernatants of PBMCs from healthy volunteers (n = 9 donors) stimulated with the fungal stimuli used for the transcriptomics (*A. fumigatus*, *C. albicans* and *R. oryzae*) compared to the unstimulated cells. Means of the Log_10_ fold-changes were calculated, and the data were clustered using hierarchical clustering on both axes (Euclidean distance, average linkage). (D) Heatmap of the expression of corresponding cytokine genes to the cytokines measured in (C) extracted from the RNA-Seq dataset of PBMCs stimulated *A. fumigatus*, *C. albicans* and *R. oryzae* for 4 h and 24 h. The Log_2_FC compared to unstimulated cells is shown, with * indicating a corrected *p-*value of < 0.05 (E) Heatmap of transcriptional expression of interferon-related genes extracted from the RNA-Seq dataset of PBMCs stimulated with *A. fumigatus*, *C. albicans,* and *R. oryzae* for 4 h and 24 h compared to unstimulated cells (RPMI); the color indicates the Log_2_FC (Shades of red indicate upregulation, while shades of green downregulation, see legend; the white * indicates adjusted *p*-value < 0.05). (F) Viability of PBMCs (n = 4) assessed by Annexin-V and PI staining after 24 h stimulation with *A. fumigatus*, *C. albicans,* and *R. oryzae*, as well as positive controls for apoptosis (Etoposide, 50 μM) and necrosis (Heat-killing of cells for 1 min at 65 °C) or negative control unstimulated cells in RPMI medium. The percentage apoptotic cells gated on the singlet, CD45^+^ cells are visualized by Annexin-V positive staining, while the percentage of necrotic cells gated on the singlet, CD45^+^ cells are visualized by Annexin-V and PI positive staining. (For interpretation of the references to color in this figure legend, the reader is referred to the web version of this article.)

To functionally compare the host response between the three fungal species, differences in the capacity of the three fungi to induce oxidative burst were evaluated in term of reactive oxygen species (ROS) production by the phagocytic NADPH oxidase complex. The Pentose Phosphate Pathway (PPP) is crucial for both fueling NADPH oxidase with NADPH to produce superoxide and for activating the cellular antioxidant systems [8], [50]. On a transcriptional level, a significant upregulation of *NCF1*, encoding the p47 phox subunit of the NADPH oxidase complex was observed after 4 h stimulation with *R. oryzae* and at both time points for *C. albicans*. *R. oryzae* significantly up-regulated some PPP enzymes. After 24 h stimulation, both *C. albicans* and *A. fumigatus* significantly up-regulated the *NCF2*, coding for the p67 phox subunit, while *R. oryzae* not only significantly down-regulated *NCF2*, but also all the other NADPH oxidase 2 subunits (Fig. 4A), as well as key enzymes of the PPP (*PGLS, TKT,* and *RPIA*). On a functional level, *C. albicans* most potently induced oxidative burst followed by *A. fumigatus*, while *R. oryzae* failed to induce any ROS release (Fig. 4B).

As cytokine and chemokine signaling were the key pathways induced as a core transcriptional response against *A. fumigatus*, *C. albicans*, and *R. oryzae,* the induction of cytokine and chemokines was further validated by systematically profiling the common features and differences in the cytokine response to these diverse fungi. PBMCs were stimulated with the three fungal species. In addition, swollen conidia were generated from *A. fumigatus* and *R. oryzae* spores*,* and hyphae from *C. albicans* yeast. Additional strains of the three fungal species were also assessed (Supplementary Fig. 1A).

In response to all the fungal species PBMCs released the proinflammatory cytokines MIP1α, TNF, IL-1β, GM-CSF, and IL-6 and the anti-inflammatory cytokine IL-1Ra. This is in line with the upregulation of the correspondent genes, with the exception of *IL1RN* for *R. oryzae* stimulation (Fig. 4D). High level of the abovementioned cytokines found also in supernatants from stimulation with diverse morphologies and strains of *A. fumigatus*, *C. albicans* and *R. oryzae* (Supplementary Fig. 1A). Apart from MIP1α, which was released in response to all the three fungi, production of the above-mentioned cytokines was higher in response to *C. albicans* and *R. oryzae* than *A. fumigatus* (Fig. 4C). IFNγ was also released in response to all fungi, albeit at low level in response to *A. fumigatus* (Fig. 4C), which may relate to its poor transcriptional induction (Fig. 4D).

Interestingly, the chemokine IFN-gamma-inducible protein 10 (IP-10, also known as CXCL10) which is abundantly produced in the course of pulmonary viral infections [4] was released by unstimulated cells, but protein levels markedly decreased in PBMCs exposed to the three fungi (Fig. 4C, Supplementary Fig. 1A).

More detailed pathway visualization and descriptions of the pathways mentioned above can be found in the related Data in Brief article [20].

#### 2.3.1 Antifungal response pathways

After identification of the genes and pathways that dictate the core transcriptional response to *A. fumigatus, C. albicans, and R. oryzae,* genes exclusively regulated by each of the fungi were investigated in more detail (Fig. 2A) to identify fungal specific transcriptional responses in cytokine signaling, pattern recognition, and metabolic pathways. A detailed overview of the species-specific induced genes and enriched pathways can be found in the related Data in Brief article [20].

Pathway enrichment analysis of the protein-coding genes specifically up-regulated after *C. albicans* stimulation confirmed *type I interferon signaling* (4 h: *p* = 3.21 × 10^-30^, GO; 24 h: *p* = 1.33 × 10^-19^ Reactome) as unique for the response to *C. albicans*. It has been previously shown that the type I IFN pathway is a characteristic transcriptional signature of anti-*Candida* host defense when compared to bacteria [95]. The comparison of the transcriptional response to *A. fumigatus* and *R. oryzae* underscores that the type I IFN pathway is also a characteristic cytokine signaling signature of anti-*Candida* host defense even when compared to other fungi (Fig. 4E).

Overall a limited transcriptional (Fig. 1A, B, E) and cytokine (Fig. 4C, D) in response to *A. fumigatus* was observed, which can be explained by the fungal properties. Structural components of the conidia, particularly hydrophobins [1], [79] and melanin [83], [98], mask pathogen associated molecular patterns (PAMPs) and thereby mediate immune evasion.

The host transcriptional response to *R. oryzae* is divergent from the other two fungi, in terms of the number of differentially regulated genes, and of the pathways modulated. The sheer amount of differentially expressed genes by *R. oryzae* was 21-fold (4 h) and 19-fold (24 h) higher compared to *A. fumigatus,* and 9-fold (4 h) and 3-fold (24 h) higher compared to *C. albicans* respectively. Pathway enrichment analysis of the genes up-regulated at both time points (4 h and 24 h) revealed significant enrichment of pathways relevant to ribosomal function, endoplasmic reticulum (ER) stress and RNA processing.

Stimulation with the three different fungal species did not impact cell viability (Fig. 4F, Supplementary Fig. 1).

### 2.4 Pathways involving pattern recognition receptors

**Fig. 5:**
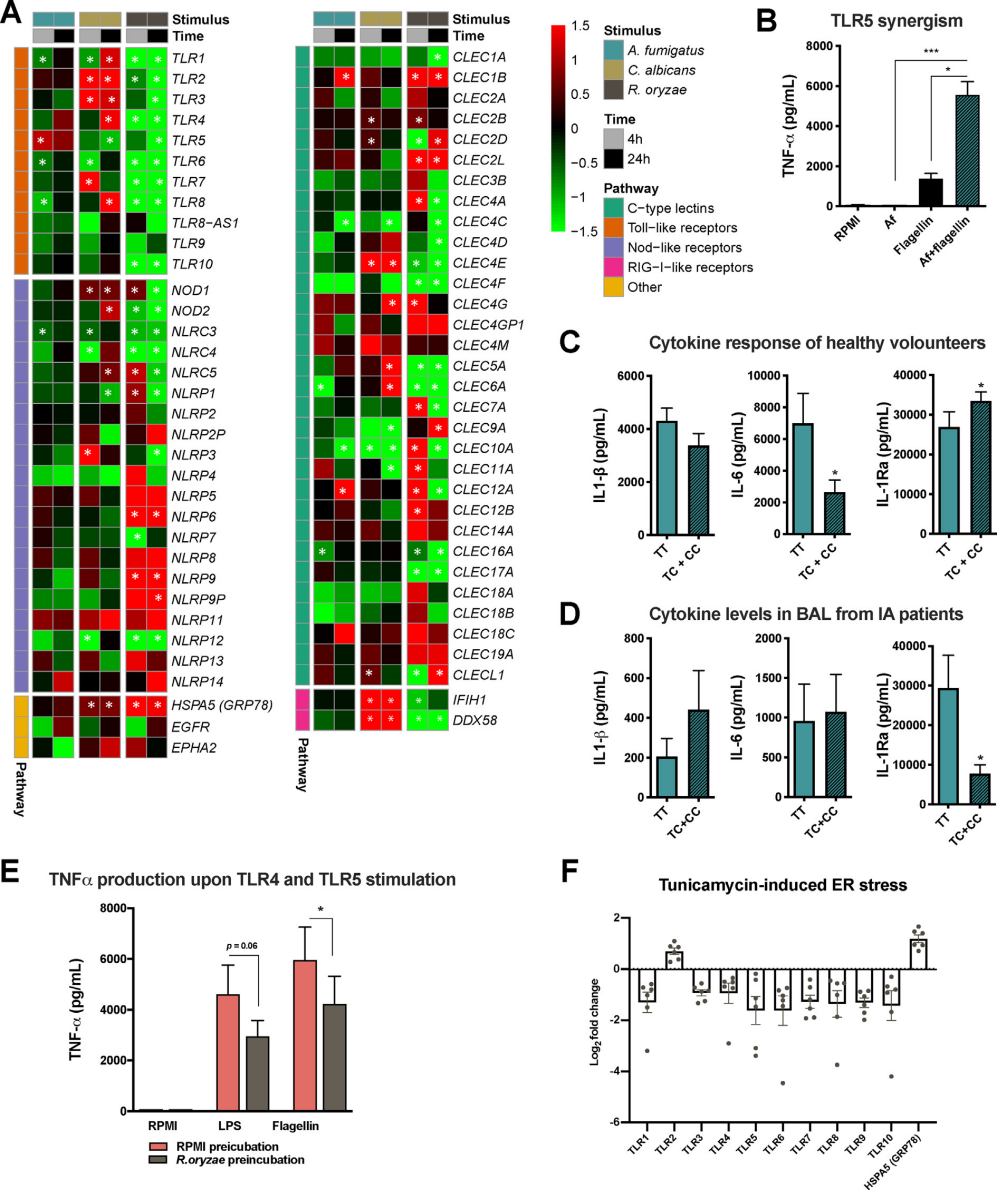
Regulation of Pattern Recognition Receptors and species-specific features: TLR5 in *A. fumigatus*-induced cytokine production and *R. oryzae*-induced downregulation of TLR signaling (A) Heatmap of transcriptional expression of Toll-like receptors (TLR), C-type lectin receptors (CLR) and NOD-like receptors (NLR) family members and GRP78 extracted from the RNA-Seq dataset of PBMCs stimulated with *A. fumigatus*, *C. albicans* and *R. oryzae* for 4 h and 24 h compared to unstimulated cells (RPMI); the color indicates the Log_2_FC (Shades of red indicate upregulation, while shades of green downregulation, see legend; the white * indicates adjusted *p*-value < 0.05). (B) TNF release in culture supernatants of PBMCs from healthy volunteers stimulated for 24 h with *A. fumigatus* V05-27 conidia (1 × 10^7^/mL), flagellin (1 µg/mL), or flagellin following 1 h of pre-incubation with *A. fumigatus* conidia; all stimulations were in the presence of 10% human serum. Data are represented as mean ± SEM and means were compared using the Kruskal Wallis test with Dunn’s multiple comparison test, *p-*values of statistical tests are shown within the graphs (n = 12; *p < 0.05; ***p < 0.001). (C) IL-1β, IL-6, IL-1Ra release in supernatants of PBMCs from healthy volunteers after stimulation for 24 h with *A. fumigatus* V05-27 heat-killed conidia (1 × 10^7^/mL). The cytokine responses were compared by stratifying for the homozygous or heterozygous presence of the C allele for *TLR5* variant (rs5744174; n = 12 TT, n = 36 TC + CC). Data are represented as mean ± SEM and means were compared using the Mann Whitney *U* test, *p-*values of statistical tests are shown within the graphs (*p < 0.05). (D) Concentrations of IL-1β, IL-6, IL-1Ra measured in BAL samples of IPA patients, which were stratified according to the genotypes for the *TLR5* variant (rs5744174; n = 11 TT, n = 8 TC + CC). Data are presented as mean ± SEM. (E) TNF release in the culture supernatant of PBMCs from healthy volunteers (n = 6 donors) pre-incubated with *R. oryzae* (PFA fixed, 1 × 10^7^/mL) for 2 h in the presence of 10% serum and subsequently re-stimulated with 10 ng/mL *E. coli* LPS (TLR4 agonist) and 1ug/mL Flagellin (TLR5 agonist) (mean ± SEM). *p < 0.05, Wilcoxon signed-rank test. (F) Expression of TLR1-10 and GRP78 quantified by qRT-PCR in PBMCs from healthy volunteers (n = 6 donors) under Tunicamycin-induced ER stress for 4 h, as compared with untreated cells. Log-fold changes are expressed as the ratio of gene expression, after normalization to β-actin. (For interpretation of the references to color in this figure legend, the reader is referred to the web version of this article.)

The initiation of the antifungal host response is mediated through the recognition of PAMPs by pattern recognition receptors (PRRs) of the host cells [100]. Comparative transcriptional analysis of PRR expression in PBMCs stimulated with the three fungal species revealed profound differences and peculiarities. *CLEC7A*, encoding the dectin-1 receptor that plays a key role in antifungal immunity, was only significantly up-regulated by *R. oryzae* after 4 h, but not by other fungi at both time points. *C. albicans* induced expression of various C-type lectin receptors (CLRs; *CLEC2B, CLEC2D, CLEC4E, CLEC4G, CLEC5A,* and *CLEC6A*). Interestingly, the nucleotide oligomerization domain-like receptors (NLRs) *NOD2* and *NLRP3* stand out as uniquely up-regulated by *C. albicans* and not by the other fungi*,* although they also play a key role in modulating host responses against *A. fumigatus* [22], [43]. Likewise, *TLR2* and *TLR4* were selectively up-regulated by *C. albicans*. The fact that expression of the intracellular nucleic acid-recognizing Toll-like receptors (TLRs; *TLR3, TLR7,* and *TLR8*), NLR (*NOD2*), together with the RIGi/MDA5 helicases *IFIH1* and *DDX58* are specifically induced by *C. albicans* further underscores that recognition of this fungus induces PRR pathways that drive type I IFN signaling (Fig. 5A).

Noteworthy, *R. oryzae* stimulation induced an early selective upregulation of some CLRs (*CLEC4A, CLEC7A, CLEC10A, CLEC11A, CLEC12A*), which are thereafter down-regulated after 24 h. In addition, the NLRs *NLRP6* and *NLRP9* were specifically up-regulated by *R. oryzae*. The receptor that mediates invasion of *R. oryzae* in endothelial host cells, GRP78 [65], also a regulator of ER stress and of the unfolded protein response (UPR) [61], was significantly up-regulated at both time points in response to *R. oryzae* and, less potently, to *C. albicans* (Fig. 5A). *GRP78* was expressed in several of the pathways that were specifically up-regulated in response to *R. oryzae*. On the contrary, another receptor involved in host tissue invasion for *Rhizopus* species, epidermal growth factor receptor (EGFR) [3], was not significantly modulated by *R. oryzae* (Fig. 5A).

Compared to the other two fungi, *A. fumigatus* stimulation did not induce expression of PRRs in PBMCs; except for *TLR5* (at 4 h), *CLEC1B* and *CLEC12A* (at 24 h). While *CLEC1B* and *CLEC12A* were not specific for *A. fumigatus*, *TLR5* was the only PRR gene specifically up-regulated by *A. fumigatus* stimulation*,* while down-regulated in response to 24 h stimulation with *C. albicans* and *R. oryzae* (Fig. 5A). Possible links between TLR5 and host defense against *A. fumigatus* have been demonstrated [88]. To validate the increased *TLR5* expression in response to *A. fumigatus*, PBMCs were pre-incubated with heat-killed *A. fumigatus* conidia to induce *TLR5* expression prior to stimulation with the TLR5 ligand flagellin. The Flagellin/TLR5-induced TNF response was significantly enhanced upon previous exposure to *A. fumigatus*, which does not induce abundant TNF responses itself (Fig. 5B). To determine whether TLR5 also plays a functional role in immune responses to *A. fumigatus,* common genetic variations in *TLR5* were analyzed for their impact on *A. fumigatus*-induced cytokine responses in a functional genomics cohort [43], [76]. The non-synonymous SNP rs5744174 (Phe616Leu) in the TLR5 ectodomain, which abrogates flagellin-induced signaling [92], has been associated with urinary tract infections [47] and hepatitis B infection [113], but it never been studied in the context of *A. fumigatus* infection. A reduced capacity to release IL-6 and an increased IL-1Ra release was observed in *A. fumigatus-*stimulated PBMCs of individuals carrying the minor allele (C) (Fig. 5C). Cytokine levels in bronchoalveolar lavage (BAL) samples from hematopoietic stem cell transplant patients (HSCT) with invasive aspergillosis (IA) were stratified based on the *TLR5* (rs5744174) donor genotypes. Patients carrying the C allele showed a trend towards increased IL-1β and decreased IL-1Ra BAL levels compared to carriers of the homozygous reference TT genotype (Fig. 5D) suggesting that this polymorphism (rs5744174) influences pulmonary inflammation during aspergillosis. These data, collectively, suggest that TLR5 and genetic variation in the *TLR5* gene differentially influences cytokine response to *A. fumigatus* and pulmonary inflammation in IA patients. In line with our data, *TLR5* is up-regulated in THP1 cells exposed to *A. fumigatus,* and TLR5 neutralization was observed to impair conidial killing [89]. Other SNPs in the *TLR5* coding region have been associated with an increased risk of aspergillosis, for example, insertion of a STOP codon (Arg392Ter; rs5744168) increased the risk of aspergillosis in HSCT patients [45]. This supports the notion that, TLR5 should be considered as a candidate gene for susceptibility to IA in at-risk patients. Future studies are also warranted to elucidate the *A. fumigatus* ligand that TLR5 recognizes.

Strikingly, many PRRs, in particular TLR genes were significantly down regulated upon *R. oryzae* stimulation (Fig. 5A). Concordantly, also the *Toll-like receptor-signaling pathway* was down-regulated (*p* = 7.32 × 10^-4^, WikiPathways). In line with the reduced expression, PBMCs exposed to *R. oryzae* demonstrate decreased responsiveness to subsequent stimulation with the TLR4 ligand LPS or the TLR5 ligand flagellin (Fig. 5E). The mechanisms driving the downregulation of TLR, signaling pathway, and cytokine genes warrants further in-depth investigation. Endoplasmic reticulum (ER) stress responses, with subsequent ER-specific mRNA degradation and reduced influx of nascent proteins into the ER lumen can result in massive gene downregulation [23], [39], [61], leading to a reduced expression of crucial host defense pathways. Stimulation of PBMCs with *R. oryzae* significantly induced genes related to the unfolded protein response and cellular response to stress, including the key player GRP78 (Fig. 5A). Similar to *R. oryzae*, PBMCs stimulated with the ER-stress inducer tunicamycin demonstrated a downregulation of TLR gene expression, with the exception of *TLR2,* and strongly up-regulated *GRP78* (Fig. 5F). These data underscore a potential connection between ER-stress responses and downregulation of key immune receptors such as TLRs.

#### 2.4.1 Metabolic rewiring of host cells: *R. oryzae* induces a dysfunctional glycolysis

Innate immune responses are energy-demanding processes and consequently involve reprogramming of cellular metabolism to meet energy demand [17]. The recognition of PAMPs induces distinct metabolic programmes in monocytes varying on the activated PRR pathway [54]. Recent works have shown rewiring of host metabolism and pointing out a crucial role for glucose metabolism in response to *C. albicans* [33], [105] and *A. fumigatus* [41]. Even though dysregulated glucose homoeostasis (e.g. hyperglycemia or diabetic ketoacidosis) increases risk for mucormycosis [10], the host metabolic response to *R. oryzae* has not been studied in detail. Moreover, effects of different fungal species on host metabolism have not been compared so far.

**Fig. 6:**
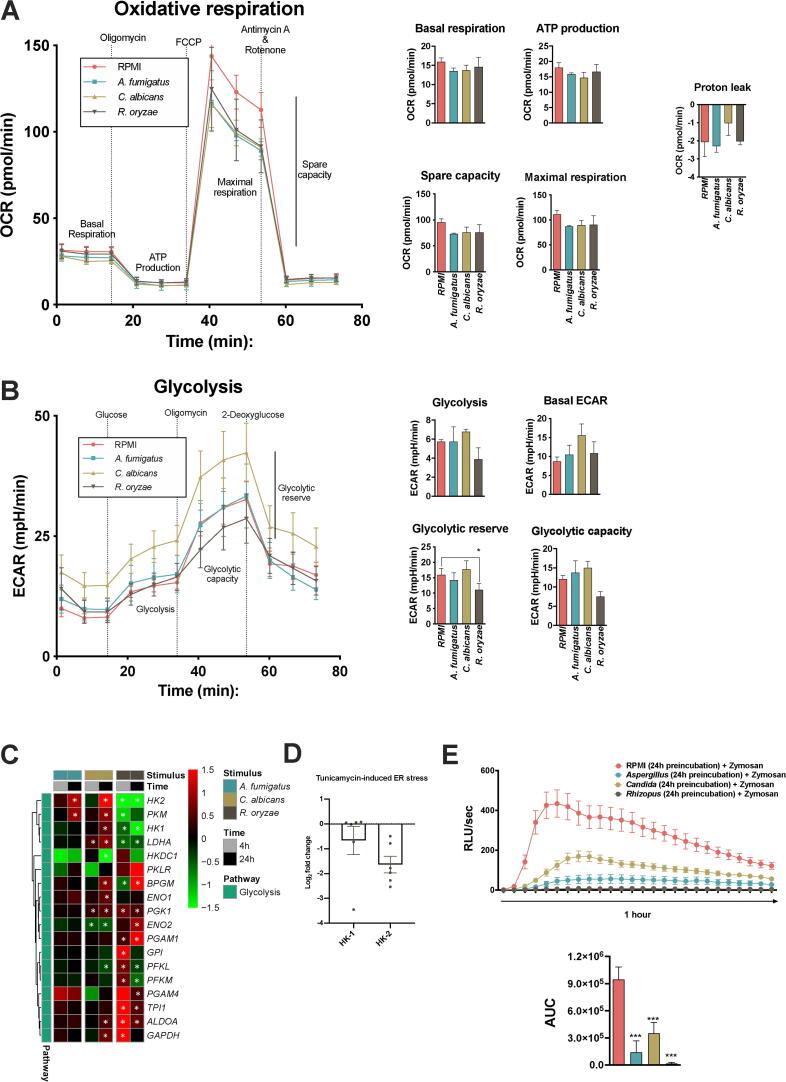
Comparative metabolic shift of host cells upon fungal stimulation: *R. oryzae* induces a dysfunctional glycolysis (A-B) After 12 h of incubation with *A. fumigatus*, *C. albicans* and *R. oryzae*, PBMCs from healthy volunteers were harvested and Oxygen Consumption Rate (OCR) (A) and Extracellular Acidification Rate (ECAR) (B) were measured by Seahorse XF e96; Basal respiration, ATP production, Maximal Respiration, Spare Respiration Capacity (SRC), Proton (H^+^) leak (A) and Basal ECAR, Glycolysis, Glycolysis, Glycolytic reserve (B) are represented (mean ± SEM, n = 3 donors, with 4–5 technical quadruplicates per donor, Student’s *t* test between *R. oryzae*- stimulated and unstimulated cells). (C) Heatmap of transcriptional expression of glycolytic genes extracted from the RNA-Seq dataset of PBMCs stimulated with *A. fumigatus*, *C. albicans* and *R. oryzae* for 4 h and 24 h compared to unstimulated cells (RPMI); the color indicates the Log_2_FC (Shades of red indicate upregulation, while shades of green downregulation; the white * indicates a corrected *p-*value of < 0.05, see legend. (D) Expression of HK1 and HK2 measured by qRT-PCR in PBMCs from healthy volunteers (n = 6 donors) under Tunicamycin-induced ER stress for 4 h, as compared with untreated cells. Log-fold changes are expressed as the ratio of gene expression, after normalization to β-actin. (E) ROS production measured in PBMCs from healthy volunteers pre-incubated with *A. fumigatus*, *C. albicans* and *R. oryzae* for 12 h in the presence of 10% serum and then re-stimulated with serum opsonized Zymosan. Measurements (ROS induction, RLU/sec) were taken within 1 h in intervals of 2.23 min and are reported as Area under curve (AUC) (mean ± SEM, n = 3 donors, with 4–5 technical quadruplicates per donor, One way ANOVA test with Dunnett’s multiple correction test). (For interpretation of the references to color in this figure legend, the reader is referred to the web version of this article.)

The significant upregulation of OXPHOS genes from each of the mitochondrial complexes after 4 h and enrichment of the *respiratory electron pathway (OXPHOS)* (*p* = 4.6 × 10^-9^, WikiPathways) suggested that *R. oryzae* stimulation specifically induces oxidative metabolism. To validate increased OXPHOS activity, PBMCs were stimulated with *A. fumigatus*, *C. albicans,* or *R. oryzae* and subsequently the oxygen consumption rate (OCR) and extracellular acidification rate (ECAR) were analyzed. In contrast to the gene expression, OCR in host cells was similar in cells stimulated by the three different fungal species (Fig. 6A). Unexpectedly, when glycolytic capacity was measured, a significant reduction was observed only in *R. oryzae*-stimulated PBMCs (Fig. 6B). This correlated with a significantly decreased transcription of the rate-limiting glycolysis enzymes hexokinase-1 (*HK1*), hexokinase-2 (*HK*2), phosphofruttokinase-1 (*PFKL*, *PFKM*), and the terminal enzyme in anaerobic glycolysis lactate dehydrogenase (*LDHA*) in response to *R. oryzae* stimulation, but not by the other fungal species (Fig. 6C). Interestingly, the downregulation of *HK2* was also found after 4 h of tunicamycin treatment in PBMCs (Fig. 5D), suggesting a role for ER-stress response induced by *R. oryzae*.

Glucose metabolism, in particular generation of the NAPDH *via* the PPP, is crucial for the generation of NADPH-oxidase-derived ROS in monocytes in response to microbial stimuli [44]. When comparing the direct fungal-induced ROS response, a defective *Rhizopus*-induced ROS-production was observed (Fig. 4B). To investigate the impact of the host metabolic changes induced by diverse fungal species on ROS production, PBMCs were exposed to *A. fumigatus*, *C. albicans,* or *R. oryzae* and subsequently zymosan-induced oxidative burst was quantified. Pre-incubation with either *A. fumigatus* or *C. albicans* slightly attenuated the capacity of PBMCs to induce an oxidative burst in response to Zymosan (Fig. 6E), which may correspond to the NADPH consumed to mount an oxidative burst in response to the pathogen by itself (Fig. 4B). Exposure to *R. oryzae* completely abolished capacity of PBMCs to induce any ROS in response to zymosan (Fig. 6E), even though it did not induce ROS in PBMCs by itself (Fig. 4B). This is in line with the significant downregulation of genes from the NADPH oxidase complex and key PPP enzymes after *R. oryzae* stimulation (Fig. 4A).

## 3 Discussion

The primary objective of this study was to elucidate the core and species-specific transcriptional responses against the distinct opportunistic pathogenic fungi *A. fumigatus, C. albicans,* and *R. oryzae.* The identified core response consists of immune-related “usual suspect” genes, involved in cytokine signaling, regulation of chemotaxis, signaling cascades, and phagocytosis/phagosome maturation. Additional genes expressed in the core response have been less thoroughly studied in the context of fungal immunology, such as vesicle remodeling, coagulation, and lncRNAs. Validation of cytokine and chemokine responses against the three fungi revealed homogeneity for several pro-inflammatory mediators, but the responses are dominated by species-specific peculiarities. Apart from the common core response, species-specific transcriptional responses were identified to the three different fungi in PRR pathways and immunometabolism. Collectively these data underscore that the host induces unique transcriptional responses to each of the three different fungi*,* but still exhibits a defined antifungal core host response.

The investigation of the core transcriptional response to *A. fumigatus, C. albicans,* and *R. oryzae* stimulation revealed that genes related to phagosome maturation and actin dynamics, which may contribute to phagocytosis and clearance, were commonly induced in response to all three fungi. The crucial role for phagocytosis in antifungal host defense is well established [5], [36]. The upregulation of both RAB20 and RAB42 suggests a role for these RAB GTPases, which have not been previously associated with antifungal host defense. RAB42 can associate with the phagosome, but its function remains unclear [114]. RAB20 is an IFNγ regulated RabGTPase involved in regulation of phagosome maturation [80], [91]. Yet, RAB20 was demonstrated to be involved in killing of engulfed *E. coli* and macrophage phagolysosome acidification [118]. Interestingly, we showed that genetic variation in close vicinity of the phagocytosis/phagosome maturation-associated genes *ASGR2, LAMP1, RAB42, GEM*, *ATP6V1B2,* and *RAB20* are associated with susceptibility to candidiasis. To explore the potential of SNP functionality in the context of fungal diseases further investigation is required. So far phagocytosis has been often studied on a species-specific basis: it has been shown that *A. fumigatus* [2], [24]*, C. albicans* [9], [110], and *R. oryzae* [5], [24] have unique features that impair phagosome maturation. Our data provides a rationale to study phagocytosis as a common denominator in antifungal host defense, which can be exploited to design immunotherapies potentially applicable to diverse fungal infections. This study highlights a role of IFNγ, which has been shown to improve fungal killing of *C. albicans* [21], [99] and *A. fumigatus* [7]. Interestingly, IFNγ induces *RAB20* and its endosomal association in macrophages [80], which points to RAB20 as a potential target *via* which IFNγ mediates its positive effects on fungal killing. Assuming that RAB20 is a key player in phagocytosis, another immunotherapeutic strategy capable of improving phagocytosis is Vitamin D3, which can improve the antimicrobial capability of macrophages [25], [63], [77], [94] and, interestingly, also induces *RAB20* [104].

Functional validation of the common cytokine signaling signature revealed that MIP1α and IL-1Ra were released in response to all three fungi. These two cytokines could, therefore, potentially be exploited as a core antifungal immunotherapy. MIP1α is currently being tested in the cancer field [90]. IL-1Ra, by competitive binding of IL-1 type 1 receptor (IL-1R), can be an immunomodulatory strategy for fungal infections where disease and pathology are the result of inflammation-driven collateral damage (e.g. VVC, pulmonary aspergillosis, or influenza-associated pulmonary aspergillosis). Recombinant IL-1Ra is already in clinical use (Anakinra/KINERET) and has been proven to reduce inflammation in murine infection models mimicking candidiasis [16] and aspergillosis [42].

A comparative transcriptional approach coupled with functional validations, offers the possibility to investigate differences in key events in antifungal host defense in term of cytokine release, pathogen recognition, and metabolic remodeling between the three pathogenic fungal species. While investigating the fungal-specific cytokine signatures, our study confirmed a previously identified key player in the response to *C. albicans.* Type I IFN response related genes were specifically induced in response to *C. albicans* and not by the other fungi. While a previous study identified type I IFN signaling as *C. albicans*-specific in comparison to the response to bacteria [95], our research pinpoints that this pathway still is specific for the response to *C. albicans*, even when compared to other opportunistic pathogenic fungi. Strategies that modulate type I IFN pathway could be auspicious targets for candidiasis-directed immunotherapy. In support of this, experimental systemic candidiasis models showed protective neutrophil recruitment dependent on the type I IFN receptor IFNAR [30]. However, given that type I IFN also has been observed to promote immunopathology [69], careful evaluation of their use as an immunotherapeutic approach is warranted.

As for the metabolic remodeling of host cells, *R. oryzae* uniquely influences cell metabolism by causing a dysfunctional glycolysis: PBMCs exposed to *R. oryzae* exhibited a reduced glycolytic rate, which was associated with reduced expression of the rate-limiting glycolysis enzymes *HK1*, *HK2*, *PKM, PFKM*, *PFKL*, and *LDHA*. In contrast, an upregulation of the OXPHOS genes, a possible compensatory response, was observed. We hypothesized that the glycolytic shutdown in response to *R. oryzae* stimulation may additionally explain why poorly controlled diabetes mellitus and obesity are risk factors for mucormycosis [28], [40], [93], [97]. Together with the high blood glucose levels and diabetic ketoacidosis (DKA) both of which induce GRP78 overexpression [5], [65], host immune cells reduce glycolysis in response to *R. oryzae* by downregulating key glycolysis genes. This may worsen diabetic patients’ glucose homeostasis, which is already impaired due to the inadequate regulation of glycolysis by insulin [18], [112]. In light of the altered glucose metabolism in response to *R. oryzae*, the glucose-lowering, insulin-sensitizing agent metformin, which promotes glycolysis [81] could be a promising therapeutic approach to change immunometabolism. In line with this, metformin treated obese flies prior to systemic *Rhizopus* infection show improved survival [93].

Despite the fact that we identified many pathways to be modulated in response to the fungal pathogens, only a fraction could be validated in the current study. We prioritized genes and pathways that may serve as potential targets for immune-directed therapy. Additionally, many interesting pathways emerged from this comparative transcriptomic analysis that warrant validation in future studies. Our study revealed many non-protein coding RNAs (lncRNAs) in the core fungal host response. However, whether and how these lncRNAs play a role in antifungal host defense remains to be elucidated. Many pathways induced by *R. oryzae* are associated to ER stress [34], [116] whose induction in PBMCs lead to a downregulation of most TLR genes and an upregulation of *GRP78*, according to our results. GRP78 (HSPA5), the host receptor for *R. oryzae,* [65], is a key regulator of these mechanisms [61] and was among the genes enriched cellular stress response and UPR pathways. It is tempting to speculate that engagement of GRP78 [66] by *R. oryzae* may play a role for the activation of these pathways [58], [115].

Another aspect that is of clinical interest is whether host characteristics (e.g. age, gender, diabetes score) influence the core and species-specific antifungal responses. Large cohort studies have revealed drastic influences of sex, age and other donor-specific parameters on circulating concentrations of anti-inflammatory mediators and monocyte-derived cytokine release [101]. Although the current study design does not facilitate such an analysis, detailed insights in the individual-specific characteristics of the core-antifungal host response could facilitate more personalized treatment strategies.

Collectively, this study identified genes and molecular pathways that comprise the core and unique antifungal host response against *A. fumigatus, C. albicans,* and *R. oryzae*. These insights can direct new studies on mechanisms that might eventually lead to novel host-directed treatment strategies.

## 4 Methods

### 4.1 Fungal strains

Several well-described fungal strains were selected for characterization of the fungal specific transcriptional response, *A. fumigatus* (Ku80 / Af1163 / CBS144.89), *C. albicans* (ATCC MYA-3573 / UC 820) [59], and *R. oryzae (delemar)* (RA 99–880 / ATCC MYA-4621) [68]. *C. albicans* was grown from glycerol stock on Sabouraud dextrose (SD) plates. For all experiments, a single colony was grown in Sabouraud broth at 37 °C for 12 h to log phase while shaking at 120 rpm. The harvested *C. albicans* yeast were washed twice in sterile phosphate buffered saline (PBS) and counted using a Bürker hemocytometer. *A. fumigatus* and *R. oryzae* were grown from glycerol stock on potato dextrose agar for 7 days at 37 °C. Conidia were harvested in sterile PBS-tween-20 (0.1%), subsequently washed twice in sterile PBS, and counted using a Bürker hemocytometer.

*C. albicans* hyphae were generated by inoculating RPMI-1640 culture medium adjusted to pH 6.4 with hydrochloric acid with a density of 1 × 10^6^/mL *Candida* yeast and culturing overnight at 37 °C while shaking at 120 rpm. Subsequently the hyphae were washed in PBS and brought to a concentration originating from 1 × 10^5^/mL yeast for the experiments. *A. fumigatus* and *R. oryzae* swollen conidia were generated by inoculating RPMI-1640 culture medium with a density of 1 × 10^6^/mL conidia, and culturing 4 h at 37 °C while shaking at 120 rpm. Subsequently the swollen conidia were washed in PBS and brought to a concentration of 1 × 10^7^/mL for the experiments. Fungi were inactivated for 30 min in 4% paraformaldehyde (*A. fumigatus* and *R. oryzae*) or heat-inactivated (*C. albicans*). For all experiments a concentration of 1 × 10^6^/mL *C. albicans* yeast was used or 1 × 10^7^/mL *A. fumigatus* or *R. oryzae* conidia.

### 4.2 Healthy volunteers and patients

For RNA-Seq experiments buffy coats were obtained from anonymized healthy donors after written consent (Sanquin Blood Bank, Nijmegen, the Netherlands). For validation experiments blood was similarly obtained from buffycoats or collected from healthy volunteers by venous blood puncture after informed consent was obtained. All experiments were performed and conducted in accordance to Good Clinical practice, the Declaration of Helsinki, and the approval of the Arnhem-Nijmegen Ethical Committee (nr.2010/104). For the validation of the findings related to several genes, healthy individuals that were recruited in the 200FG cohort study (www.humanfunctionalgenomics.org) [43], [76] participated in the study. A total of 506 hematological patients of European ancestry undergoing allogeneic hematopoietic stem-cell transplantation at Instituto Português de Oncologia, Porto, and at Hospital de Santa Maria, Lisbon, were enrolled in the IFIGEN study between 2009 and 2015. Of these, 483 had available donor and recipient DNA samples and patient-level data. Patients or their representatives provided written informed consent prior to inclusion in the study. The demographic and clinical characteristics of the patients can be found in [43]. One-hundred-eleven cases of probable/proven IPA were identified according to the 2008 criteria from the European Organization for Research and Treatment of Cancer/Mycosis Study Group (EORTC/MSG). Twenty-three patients were excluded from the study based on the “possible” classification of infection. Approval for the IFIGEN study was obtained from the SECVS (no. 125/014), the Ethics Committee for Health of the Instituto Português de Oncologia - Porto, Portugal (no. 26/015), the Ethics Committee of the Lisbon Academic Medical Center, Portugal (no. 632/014), and the National Commission for the Protection of Data, Portugal (no. 1950/015).

### 4.3 PBMC-isolation

Venous blood was drawn from healthy controls and transferred into 10 mL EDTA tubes. Subsequently, PBMCs were isolated as was described previously [96]. In short, the venous blood was diluted in PBS (1:1) before Ficoll gradient centrifugation was performed (Ficoll-Paque Plus; GE healthcare, Zeist, The Netherlands). PBMCs were washed twice in PBS and resuspended in RPMI 1640 culture medium Dutch Modification (Gibco®; Thermo Fisher, Waltham, Massachusetts, USA), supplemented with pyruvate (1 mM, Gibco®), glutamax (2 mM, Gibco®), and gentamicin (50μg/mL, Centafarm, Etten-Leur, the Netherlands). Cells were counted using a particle counter (Beckmann Coulter, Woerden, The Netherlands) after which, the concentration was adjusted to 5 × 10^6^/mL.

### 4.4 RNA-Seq

PBMCs (n = 8 donors) were stimulated in 6 well plates (1 × 10^7^ cells/well) with 1 × 10^7^/mL *A. fumigatus* (Ku80, PFA fixed) 1 × 10^6^/mL *C. albicans* (UC820, heat killed), 1 × 10^7^/mL *R. oryzae* (RA 99–880, PFA fixed), or were left unstimulated for 4 h or 24 h at 37 °C with 5% CO_2_. After incubation, the supernatant was removed and the cells were lysed and RNA was isolated using the mirVANA RNA isolation kit (Applied Biosystems) according to the protocol supplied by the manufacturer, before RNA-Seq was performed. All samples were analyzed and profiled in one batch. Before data analysis, batch effect was carefully checked. From the PCA analysis, all the samples were well separated by time (1st PCA) and by stimulation (2nd PCA). No batch effect was detected. The RNA-Seq analysis of this dataset was performed as previously described [62]. Briefly, sequencing reads were mapped to the human genome using STAR (version 2.3.0) [32]. The aligner was provided with a file containing junctions from Ensembl GRCh37.71. HTSeq-count of the Python package HTSeq (version 0.5.4p3) was used (the HTSeq package, (http://www-huber.embl.de/users/anders/HTSeq/doc/overview.html) to quantify the read counts per gene based on annotation version GRCh37.71, using the default union-counting mode. After quality control 2 donors stimulated for 4 h and three donors stimulated for 24 h with R. oryzae needed to be excluded from the analysis. Differentially expressed genes were identified by statistics analysis using DESeq2 package from bioconductor [67]. The statistically significant threshold (FDR *p* ≤ 0.05 and Fold Change ≥ 2) was applied. The data sets generated from this study have been deposited in GEO Series accession number GSE162746 (https://www.ncbi.nlm.nih.gov/geo/query/acc.cgi?acc=GSE162746).

### 4.5 Identification of core and specific host response using Venn diagram analysis

Genes were sorted based on a significance threshold of a corrected *p-*values < 0.05 and a Log_2_FC > 1 for up-regulated genes or a Log_2_FC of < −1 for down-regulated genes (Fig. 1A-C red genes). Within this list of differentially regulated genes by the 3 fungi a VENN-analysis, using the online VENN analysis tool Venny (https://bioinfogp.cnb.csic.es/tools/venny/), genes commonly up-regulated or down-regulated (significant for all 3 fungi, same direction and a Log_2_FC of > 0.9 or < −0.9) by all three fungi were identified as the core host response. Subsequently genes were identified that were induced or down-regulated for each fungus uniquely (significant for the specific fungus and a Log_2_FC of > 0.9 or < −0.9), last the sets of genes that were commonly induced or down-regulated by at least two of the fungi were identified (significant for 2 fungi, same direction and a Log_2_FC of > 0.9 or < -0.9), subsequently proportional Venn diagrams were drawn with the eulerAPE application v2.0.3 [73], [87]. The less strict threshold to group the genes for the core and species-specific responses was selected to increase the likelihood of identifying enriched pathways in the common host response, and reducing potentially false-positive enriched pathways in the species-unique responses.

### 4.6 Pathway enrichment analysis

Pathway enrichment analysis was performed using Cytoscape software with the ClueGO v.2.5.5 and CluePedia v.1.5.5 plugin [13]. To interpret and visualize the functionally group terms in the form of gene networks and pathways we have used Reactome, Wikipathways, and Gene Ontology (GO) categories, by excluding the annotations with the IEA code (Inferred from Electronic Annotation, which are assigned automatically computationally inferred based in sequence similarity comparisons. Degree of functional enrichment was determined by sorting enriched terms based on a *p-*value threshold of < 0.05. To reduce the redundancy of GO terms we applied the GO term fusion of related terms with similar associated genes. We used GO tree intervals between levels 3 and 8 and a kappa score of 0.4. The statistical test used for the enrichment was based on a right-sided hypergeometric option. The hypergeometric test *p-*values are further corrected for multiple testing using the Benjamini Hochberg multiple testing correction [12]. For *R. oryzae* modulated genes due to the high number of modulated genes, we performed pathway analysis on the list of overlapping genes between 4 h and 24 h. The complete pathways analysis results and settings are available in the related Data in Brief article.

### 4.7 Cytokine profiling with Luminex

To validate homogeneity in cytokine responses as a common pathway, PBMCs were stimulated for 24 h with *A. fumigatus, C. albicans* and *R. oryzae* as described above, supernatants were collected after 24 h of stimulation, and stored at −20 °C till analysis. Cytokine protein levels were detected with a 25-plex human cytokine panel (LHC0009M; Thermo Fischer Scientific) according to the protocol supplied by the manufacturer. Bead counts were read on a Luminex™ MAGPIX™ instrument (Thermo Fischer Scientific).

### 4.8 ROS production

The induction of reactive oxygen species was measured by oxidation luminol (5-amino-2,3,dihydro-1,4-phtalazinedione). PBMCs (5 × 10^5^), were resuspended in HBSS and put in dark 96 well plates. Cells were exposed to HBSS + 0.5% BSA, live and heat-killed *A. fumigatus* conidia (1 × 10^7^/mL), live and heat-killed *C. albicans* conidia (1 × 10^7^/mL) or live and heat-killed *R. oryzae* spores (1 × 10^7^/mL). All stimuli were pre-opsonized with 20% human serum for 30′of incubation at 37 °C prior to stimulation. Immediately 20 µL of 1 mM luminol was added. Chemiluminescence was measured in BioTek Synergy HTreader at 37 °C for every minute during one hour at intervals of 2.23 min. The relative luminescence units per second (RLU/sec) within the area under the curve (AUC) were plotted against time and analyzed by using Graphpad Prism v5.0.

### 4.9 In vitro PBMC viability assay

To determine the effect of fungal stimuli on viability of PBMCs, apoptosis and necrosis were assessed using Annexin-V and propidium iodide (PI) staining after 24 h of stimulation with the various fungal species. The cells were transferred to a v-bottom plate, washed with PBA (PBS pH 7.4, 1% w/v bovine serum albumin), and CD45 stained was stained using anti-CD45-BV510 (clone HI30; Biolegend) for 25 min at 4 °C in the dark. After washing twice with PBA, cells were stained with FITC-conjugated Annexin-V (Biovision) for 15 min at 4 °C in the dark in the presence of 5 mM CaCl_2_. Immediately afterwards, PI (Invitrogen Molecular Probes) was added and the cells were incubated an additional 5 min at 4 °C in the dark. The cells were measured using a CytoFLEX cytometer (Beckman Coulter). Data analyses were performed in FlowJo (vX.0.7). First, CD45^+^ cells were gated to exclude fungal particles from the analysis. In the CD45^+^ population single cells were selected by subsequent SSC/FSC and FSC-H/FSC-A gating. The percentage of cells positive for only FITC-Annexin-V were designated as apoptotic and cells additionally positive for PI were designated as necrotic. Etoposide-treated (50 µM; Sigma-Aldrich) and heat-killed cells were used as positive controls for apoptosis and necrosis, respectively.

### 4.10 SNP genotyping of IA patients

Genomic DNA was isolated from whole blood of patients enrolled in the IFIGEN study using the QIAcube automated system (Qiagen). Genotyping of rs5744174 in *TLR5* was performed using KASPar assays (LGC Genomics) in an Applied Biosystems 7500 Fast Real-Time PCR system (Thermo Fisher Scientific), according to the manufacturer’s instructions.

### 4.11 GWAS on candidemia susceptibility

The GWAS on candidemia susceptibility was previously described [48]. In short, upon quality control per SNP and sample, this GWAS was performed in a cohort of 161 candidemia cases and 152 disease-matched controls of European ancestry whose demographic and clinical characteristics have been previously described [53]. DNA was genotyped using Illumina HumanCoreExome-12 v1.0 and HumanCoreExome-24 v1.0 BeadChip SNP chips. Genotypes were imputed using the human reference consortium (HRC) panel [71] using the Michigan imputation server [29]. In total, 5,426,313 SNPs were tested for disease association using Fisher’s exact test with PLINK v1.9 [84]. Detailed results and statistics can be found in Table 1.

### 4.12 BAL fluid collection

BAL specimens were collected from patients using a flexible fiberoptic bronchoscope following local anesthesia with 2% lidocaine (Xylocaine), when infection was clinically suspected. Samples were obtained by instillation of a 0.9% sterile saline solution (20 mL twice). The sampling area was determined based on the localization of lesion on chest imaging. BAL specimens with comparable recovery rates were used.

### 4.13 ELISA for cytokine in PBMCs supernatant and patient BAL

Cytokine levels were measured in the cell culture supernatants from PBMCs using commercially available ELISA assays (R&D systems) according to the protocol supplied by the manufacturer. For human cytokines, IL-1β, TNF, IL-6, and IL-1Ra were measured after 24 h of stimulation.

Cytokines were quantified in BAL samples using customized Human Premixed Multi-Analyte Kits (R&D Systems, MN, USA). All cytokine determinations were performed in duplicates, and concentrations were reported in pg/mL.

### 4.14 ER-stress induction in PBMCs and qPCR

PBMCs were incubated with 2 ug/mL tunicamycin in RPMI 1640 for 4 h. RNA was isolated using RNeasy Mini Kit (Qiagen), according to the manufacturer's instructions. For reverse transcription-quantitative PCR (RT-qPCR), isolated RNA (500 ng) was treated with DNase I (Fermentas) and subsequently transcribed into cDNA using 0.5 µg Oligo(dT)12–18 Primer, 200 U Superscript™ III Reverse Transcriptase and 40 U RNaseOUT™ Recombinant RNase Inhibitor (Thermo Fischer Scientific). RT-qPCR was performed with GoTaq® qPCR Master Mix (Promega) in a CFX96 thermocycler (Bio-Rad) and the expression levels were normalized against β-actin. All the primers used are listed in Supplementary Table 2.

### 4.15 Seahorse metabolic analysis

2 × 10^6^ PBMCs were seeded in a polypropylene tube (BD Falcon) in 2 mL of RPMI 10% serum and stimulated as previously described for RNA-Seq for 12 h at 37 °C, 5% CO_2_. After that, 4 × 10^5^ cells were plated to overnight-calibrated cartridges in assay medium (either DMEM supplemented with 2 mM glutamine, 11 mM glucose and 1 mM pyruvate, or DMEM supplemented with 1 mM glutamine depending on the assay [pH adjusted to 7.4]) and incubated for 1 h in a non-CO_2_-corrected incubator at 37 °C. Oxygen consumption rate (OCR) and extracellular acidification rate (ECAR) were measured using Cell Mito Stress test (for OCR) kit and the Glycolysis Stress test (for ECAR) in an XFe96 Analyzer (Seahorse Bioscience), with final concentrations of 1 µM oligomycin, 1 µM FCCP, and 1.25/2.5 µM rotenone/antimycin A for the Mito Stress test, and 11 mM glucose, 1 µM oligomycin, and 22 mM 2-DG for the Glycolysis Stress test.

### 4.16 Statistical analysis

For the RNA-Seq data and the pathways analysis statistical details can be found in the dedicated method sections. When comparing two differentially stimulated groups, or groups with different genotypes, we used Student’s t tests or the Mann-Whitney *U* test depending on the distribution of the data. ANOVA with Bonferroni’s adjustment was used to determine the statistical significance for multiple groups. Data were analyzed by GraphPad Prism version 6. Data are presented as means + SEM for all bar graphs. *p < 0.05, **p < 0.001, ***p < 0.001, and ****p < 0.0001.

## CRediT authorship contribution statement

**Mariolina Bruno:** Validation, Investigation, Data curation, Writing - original draft, Visualization. **Intan M.W. Dewi:** Investigation, Visualization. **Vicky Matzaraki:** Formal analysis, Investigation, Resources, Visualization. **Rob ter Horst:** Software, Formal analysis, Investigation, Resources, Visualization, Data curation. **Marina Pekmezovic:** Investigation, Visualization. **Berenice Rösler:** Investigation, Visualization. **Laszlo Groh:** Investigation, Visualization. **Rutger J. Röring:** Investigation, Visualization. **Vinod Kumar:** Methodology, Software, Formal analysis, Investigation, Data curation. **Yang Li:** Methodology, Software, Formal analysis, Investigation, Data curation. **Agostinho Carvalho:** Methodology, Validation, Formal analysis, Investigation, Resources. **Mihai G. Netea:** Conceptualization, Resources, Supervision, Project administration, Funding acquisition. **Jean-Paul Latgé:** Conceptualization, Resources, Supervision, Funding acquisition. **Mark S. Gresnigt:** Conceptualization, Methodology, Validation, Formal analysis, Investigation, Data curation, Writing - original draft, Visualization, Supervision, Project administration. **Frank L. van de Veerdonk:** Conceptualization, Supervision, Project administration.

## Declaration of Competing Interest

The authors declare that they have no known competing financial interests or personal relationships that could have appeared to influence the work reported in this paper.

## References

[1] Aimanianda V., Bayry J., Bozza S., Kniemeyer O., Perruccio K., Elluru S.R., Clavaud C., Paris S., Brakhage A.A., Kaveri S.V., Romani L., Latgé J.-P. (2009). Surface hydrophobin prevents immune recognition of airborne fungal spores. Nature.

[2] Akoumianaki T., Kyrmizi I., Valsecchi I., Gresnigt M., Samonis G., Drakos E., Boumpas D., Muszkieta L., Prevost M.-C., Kontoyiannis D., Chavakis T., Netea M., van de Veerdonk F., Brakhage A., El-Benna J., Beauvais A., Latge J.-P., Chamilos G. (2016). Aspergillus cell wall melanin blocks LC3-associated phagocytosis to promote pathogenicity. Cell Host Microbe.

[3] Alqarihi A, Gebremariam T, Gu Y, Swidergall M, Alkhazraji S, Soliman SSM, Bruno VM, Edwards JE, Filler SG, Uppuluri P, et al.. GRP78 and Integrins Play Different Roles in Host Cell Invasion during Mucormycosis. MBio (2020) 11. 10.1128/mBio.01087-20 PMC7267888 32487760

[4] Alves M.P., Schögler A., Muster R., Kronig M.-N., Kieninger E., Kopf B.S. (2013). IP-10 is selectively produced in the airways upon respiratory virus infection. Eur Respir J.

[5] Andrianaki A.M., Kyrmizi I., Thanopoulou K., Baldin C., Drakos E., Soliman S.S.M. (2018). Iron restriction inside macrophages regulates pulmonary host defense against Rhizopus species. Nat Commun.

[6] Armstrong-James D., Brown G.D., Netea M.G., Zelante T., Gresnigt M.S., van de Veerdonk F.L., Levitz S.M. (2017). Immunotherapeutic approaches to treatment of fungal diseases. Lancet Infect Dis.

[7] Assendorp E.L., Gresnigt M.S., Sprenkeler E.G.G., Meis J.F., Dors N., van der Linden J.W.M., Henriet S.S.V. (2018). Adjunctive interferon-γ immunotherapy in a pediatric case of Aspergillus terreus infection. Eur J Clin Microbiol Infect Dis.

[8] Azevedo E.P., Rochael N.C., Guimarães-Costa A.B., de Souza-Vieira T.S., Ganilho J., Saraiva E.M., Palhano F.L., Foguel D. (2015). A Metabolic shift toward pentose phosphate pathway is necessary for amyloid fibril- and phorbol 12-myristate 13-acetate-induced neutrophil extracellular trap (NET) formation. J Biol Chem.

[9] Bain J.M., Louw J., Lewis L.E., Okai B., Walls C.A., Ballou E.R., Walker L.A., Reid D., Munro C.A., Brown A.J.P., Brown G.D., Gow N.A.R., Erwig L.P., Heitman J. (2014). Candida albicans hypha formation and mannan masking of β-glucan inhibit macrophage phagosome maturation. mBio.

[10] Baldin C, and Ibrahim AS. Molecular mechanisms of mucormycosis—The bitter and the sweet. PLOS Pathog (2017) 13, e1006408. 10.1371/journal.ppat.1006408 PMC5542377 28771587

[11] Bassetti M., Mikulska M., Viscoli C. (2010). Bench-to-bedside review: therapeutic management of invasive candidiasis in the intensive care unit. Crit Care.

[12] Benjamini Y., Hochberg Y. (1995). Controlling the false discovery rate: a practical and powerful approach to multiple testing. J Roy Stat Soc: Ser B (Methodol).

[13] Bindea G, Mlecnik B, Hackl H, Charoentong P, Tosolini M, Kirilovsky A, Fridman WH, Pagès F, Trajanoski Z, and Galon J. ClueGO: a Cytoscape plug-in to decipher functionally grouped gene ontology and pathway annotation networks. Bioinformatics 25 (2009), 1091–1093. 10.1093/bioinformatics/btp101 PMC2666812 19237447

[14] Bongomin F, Gago S, Oladele RO, and Denning DW. Global and Multi-National Prevalence of Fungal Diseases—Estimate Precision. J. Fungi (2017) 3. 10.3390/jof3040057 PMC5753159 29371573

[15] Bonnet S., Duléry R., Regany K., Bouketouche M., Magro L., Coiteux V., Alfandari S., Berthon C., Quesnel B., Yakoub-Agha I. (2017). Long-term follow up of invasive aspergillosis in allogeneic stem cell transplantation recipients and leukemia patients: differences in risk factors and outcomes. Curr Res Transl Med.

[16] Borghi M., De Luca A., Puccetti M., Jaeger M., Mencacci A., Oikonomou V., Pariano M., Garlanda C., Moretti S., Bartoli A., Sobel J., van de Veerdonk F., Dinarello C., Netea M., Romani L. (2015). Pathogenic NLRP3 inflammasome activity during candida infection is negatively regulated by IL-22 via activation of NLRC4 and IL-1Ra. Cell Host Microbe.

[17] den Bossche J.V., O’Neill L.A., Menon D. (2017). Macrophage immunometabolism: where are we (Going)?. Trends Immunol.

[18] Bouché C, Serdy S, Kahn CR, and Goldfine AB. The Cellular Fate of Glucose and Its Relevance in Type 2 Diabetes. Endocr Rev (2004) 25, 807–830. 10.1210/er.2003-0026 15466941

[19] Brown GD, Denning DW, Gow NAR, Levitz SM, Netea MG, and White TC. Hidden Killers: Human Fungal Infections. Sci Transl Med (2012) 4, 165rv13-165rv13. 10.1126/scitranslmed.3004404 23253612

[20] Bruno M, Ter Horst R, Pekmezovic M, Kumar V, Li Y, Netea MG, Latgé JP, Gresnigt MS, and van de Veerdonk FL. Data of common and species-specific transcriptional host responses to pathogenic fungi. Data in Brief (2021). 10.1016/j.dib.2021.106928 PMC8039545 33850980

[21] Buddingh E.P., Leentjens J., van der Lugt J., Dik W.A., Gresnigt M.S., Netea M.G., Pickkers P., Driessen G.J. (2015). Interferon-gamma Immunotherapy in a patient with refractory disseminated candidiasis. Pediatr Infect Dis J.

[22] Campos C.F., van de Veerdonk F.L., Gonçalves S.M., Cunha C., Netea M.G., Carvalho A. (2019). Host genetic signatures of susceptibility to fungal disease. Curr Top Microbiol Immunol.

[23] Casas C. (2017). GRP78 at the centre of the stage in cancer and neuroprotection. Front Neurosci.

[24] Chamilos G., Akoumianaki T., Kyrmizi I., Brakhage A., Beauvais A., Latge J.-P. (2016). Melanin targets LC3-associated phagocytosis (LAP): a novel pathogenetic mechanism in fungal disease. Autophagy.

[25] Chandra G., Selvaraj P., Jawahar M.S., Banurekha V.V., Narayanan P.R. (2004). Effect of Vitamin D 3 on phagocytic potential of macrophages with live mycobacterium tuberculosis and lymphoproliferative response in pulmonary tuberculosis. J Clin Immunol.

[26] Clark C, and Drummond RA. The Hidden Cost of Modern Medical Interventions: How Medical Advances Have Shaped the Prevalence of Human Fungal Disease. Pathogens (2019) 8, 45. 10.3390/pathogens8020045 PMC6631793 30987351

[27] Cooper D.N. (2010). Functional intronic polymorphisms: buried treasure awaiting discovery within our genes. Hum Genomics.

[28] Corzo-León DE, Chora-Hernández LD, Rodríguez-Zulueta AP, and Walsh TJ. Diabetes mellitus as the major risk factor for mucormycosis in Mexico: Epidemiology, diagnosis, and outcomes of reported cases. Med Mycol (2018) 56, 29–43. 10.1093/mmy/myx017 28431008

[29] Das S., Forer L., Schönherr S., Sidore C., Locke A.E., Kwong A., Vrieze S.I., Chew E.Y., Levy S., McGue M., Schlessinger D., Stambolian D., Loh P.-R., Iacono W.G., Swaroop A., Scott L.J., Cucca F., Kronenberg F., Boehnke M., Abecasis G.R., Fuchsberger C. (2016). Next-generation genotype imputation service and methods. Nat Genet.

[30] del Fresno C., Soulat D., Roth S., Blazek K., Udalova I., Sancho D., Ruland J., Ardavín C. (2013). Interferon-β production via dectin-1-Syk-IRF5 signaling in dendritic cells is crucial for immunity to C. albicans. Immunity.

[31] Dewi I.M.W., Aleva F.E., Kullaya V.I., Garishah F.M., de Mast Q., van der Ven A.J.A.M., van de Veerdonk F.L. (2020). Platelets modulate IFN-γ production against candida albicans in peripheral blood mononuclear cells via prostaglandins. J Immunol.

[32] Dobin A, Davis CA, Schlesinger F, Drenkow J, Zaleski C, Jha S, Batut P, Chaisson M, and Gingeras TR. STAR: ultrafast universal RNA-seq aligner. Bioinforma. Oxf. Engl. (2013) 29, 15–21. 10.1093/bioinformatics/bts635 PMC3530905 23104886

[33] Domínguez-Andrés J, Arts RJW, ter Horst R, Gresnigt MS, Smeekens SP, Ratter JM, Lachmandas E, Boutens L, van de Veerdonk FL, Joosten LAB, et al.. Rewiring monocyte glucose metabolism via C-type lectin signaling protects against disseminated candidiasis. PLOS Pathog. (2017) 13, e1006632. 10.1371/journal.ppat.1006632 PMC5619837 28922415

[34] DuRose J.B., Scheuner D., Kaufman R.J., Rothblum L.I., Niwa M. (2009). Phosphorylation of eukaryotic translation initiation factor 2alpha coordinates rRNA transcription and translation inhibition during endoplasmic reticulum stress. Mol Cell Biol.

[35] Eades C.P., Armstrong-James D.P.H. (2019). Invasive fungal infections in the immunocompromised host: mechanistic insights in an era of changing immunotherapeutics. Med Mycol.

[36] Erwig L.P., Gow N.A.R. (2016). Interactions of fungal pathogens with phagocytes. Nat Rev Microbiol.

[37] Fréalle E., Gosset P., Leroy S., Delattre C., Wacrenier A., Zenzmaier C., Zawadzki C., Aliouat E.M., Perkhofer S. (2018). In vitro coagulation triggers anti- Aspergillus fumigatus neutrophil response. Future Microbiology.

[38] Garcia‐Vidal C., Upton A., Kirby K., Marr K. (2008). Epidemiology of invasive mold infections in allogeneic stem cell transplant recipients: biological risk factors for infection according to time after transplantation. Clin infect Dis.

[39] Gardner BM, Pincus D, Gotthardt K, Gallagher CM, and Walter P. Endoplasmic Reticulum Stress Sensing in the Unfolded Protein Response. Cold Spring Harb. Perspect. Biol. (2013) 5. 10.1101/cshperspect.a013169 PMC3578356 23388626

[40] Gleissner B., Schilling A., Anagnostopolous I., Siehl I., Thiel E. (2004). Improved outcome of zygomycosis in patients with hematological diseases?. Leukemia Lymphoma.

[41] Gonçalves S.M., Duarte-Oliveira C., Campos C.F., Aimanianda V., ter Horst R., Leite L., Mercier T., Pereira P., Fernández-García M., Antunes D. (2020). Phagosomal removal of fungal melanin reprograms macrophage metabolism to promote antifungal immunity. Nat Commun.

[42] Gresnigt M.S., Rekiki A., Rasid O., Savers A., Jouvion G., Dannaoui E. (2016). Reducing hypoxia and inflammation during invasive pulmonary aspergillosis by targeting the Interleukin-1 receptor. Sci Rep.

[43] Gresnigt M.S., Cunha C., Jaeger M., Gonçalves S.M., Malireddi R.K.S., Ammerdorffer A., Lubbers R., Oosting M., Rasid O., Jouvion G., Fitting C., Jong D.J.d., Lacerda J.F., Campos A., Melchers W.J.G., Lagrou K., Maertens J., Kanneganti T.-D., Carvalho A., Ibrahim-Granet O., van de Veerdonk F.L. (2018). Genetic deficiency of NOD2 confers resistance to invasive aspergillosis. Nat Commun.

[44] Grondman I., Arts R.J.W., Koch R.M., Leijte G.P., Gerretsen J., Bruse N., Kempkes R.W.M., ter Horst R., Kox M., Pickkers P., Netea M.G., Gresnigt M.S. (2019). Frontline science: endotoxin‐induced immunotolerance is associated with loss of monocyte metabolic plasticity and reduction of oxidative burst. J Leukoc Biol.

[45] Grube M., Loeffler J., Mezger M., Krüger B., Echtenacher B., Hoffmann P., Edinger M., Einsele H., Andreesen R., Holler E. (2013). TLR5 stop codon polymorphism is associated with invasive aspergillosis after allogeneic stem cell transplantation. Med Mycol.

[46] Hallen-Adams H.E., Suhr M.J. (2017). Fungi in the healthy human gastrointestinal tract. Virulence.

[47] Hawn TR, Scholes D, Li SS, Wang H, Yang Y, Roberts PL, Stapleton AE, Janer M, Aderem A, Stamm WE et al.. Toll-Like Receptor Polymorphisms and Susceptibility to Urinary Tract Infections in Adult Women. PLoS ONE (2009) 4. 10.1371/journal.pone.0005990 PMC2696082 19543401

[48] Jaeger M, Matzaraki V, Aguirre-Gamboa R, Gresnigt MS, Chu X, Johnson MD, Oosting M, Smeekens SP, Withoff S, Jonkers I et al.. A Genome-Wide Functional Genomics Approach Identifies Susceptibility Pathways to Fungal Bloodstream Infection in Humans. J. Infect. Dis. (2019) 220, 862–872. 10.1093/infdis/jiz206 PMC6667794 31241743

[49] Kämmer P, McNamara S, Wolf T, Conrad T, Allert S, Gerwien F, Hünniger K, Kurzai O, Guthke R, Hube B et al.. Survival Strategies of Pathogenic Candida Species in Human Blood Show Independent and Specific Adaptations. MBio (2020) 11. 10.1128/mBio.02435-20 PMC7542370 33024045

[50] Koo S, Szczesny B, Wan X, Putluri N, and Garg NJ. Pentose Phosphate Shunt Modulates Reactive Oxygen Species and Nitric Oxide Production Controlling Trypanosoma cruzi in Macrophages. Front. Immunol. (2018) 9. 10.3389/fimmu.2018.00202 PMC5820298 29503646

[51] Kullberg B.J., Arendrup M.C. (2015). Invasive candidiasis. N Engl J Med.

[52] Kumamoto C.A., Gresnigt M.S., Hube B. (2020). The gut, the bad and the harmless: Candida albicans as a commensal and opportunistic pathogen in the intestine. Curr Opin Microbiol.

[53] Kumar V., Cheng S.-C., Johnson M.D., Smeekens S.P., Wojtowicz A., Giamarellos-Bourboulis E., Karjalainen J., Franke L., Withoff S., Plantinga T.S. (2014). Immunochip SNP array identifies novel genetic variants conferring susceptibility to candidaemia. Nat Commun.

[54] Lachmandas E., Boutens L., Ratter J.M., Hijmans A., Hooiveld G.J., Joosten L.A.B. (2016). Microbial stimulation of different Toll-like receptor signalling pathways induces diverse metabolic programmes in human monocytes. Nat Microbiol.

[55] Lagunes L., Rello J. (2016). Invasive candidiasis: from mycobiome to infection, therapy, and prevention. Eur J Clin Microbiol Infect Dis.

[56] Lai C.C., Liaw S.J., Lee L.N., Hsiao C.H., Yu C.J., Hsueh P.R. (2007). Invasive pulmonary aspergillosis: high incidence of disseminated intravascular coagulation in fatal cases. J Microbiol Immunol Infect Wei Mian Yu Gan Ran Za Zhi.

[57] Latgé J.-P. (2001). The pathobiology of Aspergillus fumigatus. Trends Microbiol.

[58] Lee A.S. (2005). The ER chaperone and signaling regulator GRP78/BiP as a monitor of endoplasmic reticulum stress. Methods.

[59] Lehrer R.I., Cline M.J. (1969). Interaction of Candida albicans with human leukocytes and serum. J Bacteriol.

[60] Lewis R.E., Georgiadou S.P., Sampsonas F., Chamilos G., Kontoyiannis D.P. (2014). Risk factors for early mortality in haematological malignancy patients with pulmonary mucormycosis. Mycoses.

[61] Li J., Ni M., Lee B., Barron E., Hinton D.R., Lee A.S. (2008). The unfolded protein response regulator GRP78/BiP is required for endoplasmic reticulum integrity and stress-induced autophagy in mammalian cells. Cell Death Differ.

[62] Li Y., Oosting M., Deelen P., Ricaño-Ponce I., Smeekens S., Jaeger M. (2016). Inter-individual variability and genetic influences on cytokine responses against bacterial and fungal pathogens. Nat Med.

[63] Lim J.H.J., Ravikumar S., Wang Y.-M., Thamboo T.P., Ong L., Chen J., Goh J.G., Tay S.H., Chengchen L., Win M.S., Leong W., Lau T., Foo R., Mirza H., Tan K.S.W., Sethi S., Khoo A.L., Chng W.J., Osato M., Netea M.G., Wang Y., Chai L.Y.A. (2015). Bimodal influence of vitamin d in host response to systemic *Candida* infection—vitamin D dose matters. J Infect Dis.

[64] Lionakis M.S., Kontoyiannis D.P. (2003). Glucocorticoids and invasive fungal infections. Lancet.

[65] Liu M., Spellberg B., Phan Q.T., Fu Y., Fu Y., Lee A.S., Edwards J.E., Filler S.G., Ibrahim A.S. (2010). The endothelial cell receptor GRP78 is required for mucormycosis pathogenesis in diabetic mice. J Clin Invest.

[66] Louessard M., Bardou I., Lemarchand E., Thiebaut A.M., Parcq J., Leprince J., Terrisse A., Carraro V., Fafournoux P., Bruhat A., Orset C., Vivien D., Ali C., Roussel B.D. (2017). Activation of cell surface GRP78 decreases endoplasmic reticulum stress and neuronal death. Cell Death Differ.

[67] Love M.I., Huber W., Anders S. (2014). Moderated estimation of fold change and dispersion for RNA-seq data with DESeq2. Genome Biol.

[68] Ma LJ, Ibrahim AS, Skory C, Grabherr MG, Burger G, Butler M, Elias M, Idnurm A, Lang BF, Sone T et al.. Genomic analysis of the basal lineage fungus Rhizopus oryzae reveals a whole-genome duplication. PLoS Genet. (2009) 5, e1000549. 10.1371/journal.pgen.1000549 PMC2699053 19578406

[69] Majer O, Bourgeois C, Zwolanek F, Lassnig C, Kerjaschki D, Mack M, Müller M, and Kuchler K. Type I interferons promote fatal immunopathology by regulating inflammatory monocytes and neutrophils during Candida infections. PLoS Pathog. (2012) 8, e1002811. 10.1371/journal.ppat.1002811 PMC3406095 22911155

[70] Matzaraki V, Gresnigt MS, Jaeger M, Ricaño-Ponce I, Johnson MD, Oosting M, Franke L, Withoff S, Perfect JR, Joosten LAB et al.. An integrative genomics approach identifies novel pathways that influence candidaemia susceptibility. PloS One (2017) 12, e0180824. 10.1371/journal.pone.0180824 PMC5519064 28727728

[71] McCarthy S., Das S., Kretzschmar W., Delaneau O., Wood A.R., Teumer A. (2016). A reference panel of 64,976 haplotypes for genotype imputation. Nat Genet.

[72] Meis J.F., Chakrabarti A. (2009). Changing epidemiology of an emerging infection: zygomycosis. Clin Microbiol Infect Off Publ Eur Soc Clin Microbiol Infect Dis.

[73] Micallef L, and Rodgers P. eulerAPE: Drawing Area-Proportional 3-Venn Diagrams Using Ellipses. PLOS ONE (2014) 9, e101717. 10.1371/journal.pone.0101717 PMC4102485 25032825

[74] Munoz J., Hughes A., Guo Y. (2013). Mucormycosis-associated intracranial hemorrhage. Blood Coagul Fibrinolysis.

[75] Naglik J.R., Moyes D.L., Wächtler B., Hube B. (2011). Candida albicans interactions with epithelial cells and mucosal immunity. Microbes Infect.

[76] Netea M.G., Joosten L.A.B., Li Y., Kumar V., Oosting M., Smeekens S., Jaeger M., ter Horst R., Schirmer M., Vlamakis H. (2016). Understanding human immune function using the resources from the Human Functional Genomics Project. Nat Med.

[77] Nouari W., Ysmail-Dahlouk L., Aribi M. (2016). Vitamin D3 enhances bactericidal activity of macrophage against Pseudomonas aeruginosa. Int Immunopharmacol.

[78] Pappas P.G., Lionakis M.S., Arendrup M.C., Ostrosky-Zeichner L., Kullberg B.J. (2018). Invasive candidiasis. Nat Rev Dis Primer.

[79] Paris S., Debeaupuis J.-P., Crameri R., Carey M., Charlès F., Prévost M.C., Schmitt C., Philippe B., Latgé J.P. (2003). Conidial hydrophobins of Aspergillus fumigatus. Appl Environ Microbiol.

[80] Pei G., Schnettger L., Bronietzki M., Repnik U., Griffiths G., Gutierrez M.G., Gruenberg J.E. (2015). Interferon-γ–inducible Rab20 regulates endosomal morphology and EGFR degradation in macrophages. Mol Biol Cell.

[81] Pernicova I., Korbonits M. (2014). Metformin—mode of action and clinical implications for diabetes and cancer. Nat Rev Endocrinol.

[82] Philippidis P., Naiman J.L., Sibinga M.S., Valdes-Dapnea M.A. (1971). Disseminated intravascular coagulation in Candida albicans septicemia. J Pediatr.

[83] Pihet M., Vandeputte P., Tronchin G., Renier G., Saulnier P., Georgeault S., Mallet R., Chabasse D., Symoens F., Bouchara J.-P. (2009). Melanin is an essential component for the integrity of the cell wall of Aspergillus fumigatus conidia. BMC Microbiol.

[84] Purcell S., Neale B., Todd-Brown K., Thomas L., Ferreira M.A.R., Bender D., Maller J., Sklar P., de Bakker P.I.W., Daly M.J., Sham P.C. (2007). PLINK: a tool set for whole-genome association and population-based linkage analyses. Am J Hum Genet.

[85] Ribes J.A., Vanover-Sams C.L., Baker D.J. (2000). Zygomycetes in human disease. Clin Microbiol Rev.

[86] Richardson M. (2009). The ecology of the Zygomycetes and its impact on environmental exposure. Clin Microbiol Infect Off Publ Eur Soc Clin Microbiol Infect Dis.

[87] Rodgers P., Flower J., Stapleton G., Howse J., Goel A.K., Jamnik M., Narayanan N.H. (2010). Drawing area-proportional venn-3 diagrams with convex polygons. Diagrammatic Representation and Inference.

[88] Rødland E.K., Mattingsdal M., Olstad O.K., Øvstebø R., Kierulf P., Műller F., Frøland S.S. (2008). Expression of genes in normal human monocytes in response to Aspergillus fumigatus. Med Mycol.

[89] Rødland E.K., Ager-Wick E., Halvorsen B., Müller F., Frøland S.S. (2011). Toll like receptor 5 (TLR5) may be involved in the immunological response to Aspergillus fumigatus in vitro. Med Mycol.

[90] Schaller T.H., Batich K.A., Suryadevara C.M., Desai R., Sampson J.H. (2017). Chemokines as adjuvants for immunotherapy: implications for immune activation with CCL3. Expert Rev Clin Immunol.

[91] Schnettger L., Rodgers A., Repnik U., Lai R.P., Pei G., Verdoes M., Wilkinson R.J., Young D.B., Gutierrez M.G. (2017). A Rab20-dependent membrane trafficking pathway controls M. tuberculosis replication by regulating phagosome spaciousness and integrity. Cell Host Microbe.

[92] Sheridan J, Mack DR, Amre DK, Israel DM, Cherkasov A, Li H, Grimard G, and Steiner TS. A non-synonymous coding variant (L616F) in the TLR5 gene is potentially associated with Crohn’s disease and influences responses to bacterial flagellin. PloS One (2013) 8, e61326. 10.1371/journal.pone.0061326 PMC3623901 23593463

[93] Shirazi F, Farmakiotis D, Yan Y, Albert N, Kim-Anh D, and Kontoyiannis DP. Diet Modification and Metformin Have a Beneficial Effect in a Fly Model of Obesity and Mucormycosis. PLOS ONE (2014) 9, e108635. 10.1371/journal.pone.0108635 PMC4182538 25268492

[94] Small A, Harvey S, Kaur J, Putty T, Quach A, Munawara U, Hii C, and Ferrante A. Vitamin D promotes anti-microbial activity of macrophages via Complement Receptor Immunoglobulin. J. Immunol. (2019) 202, 126.34-126.34.

[95] Smeekens S.P., Ng A., Kumar V., Johnson M.D., Plantinga T.S., van Diemen C., Arts P., Verwiel E.T.P., Gresnigt M.S., Fransen K. (2013). Functional genomics identifies type I interferon pathway as central for host defense against Candida albicans. Nat Commun.

[96] Smeekens S.P., Gresnigt M.S., Becker K.L., Cheng S.-C., Netea S.A., Jacobs L., Jansen T., van de Veerdonk F.L., Williams D.L., Joosten L.A.B. (2015). An anti-inflammatory property of Candida albicans β-glucan: Induction of high levels of interleukin-1 receptor antagonist via a Dectin-1/CR3 independent mechanism. Cytokine.

[97] Spellberg B., Edwards J., Ibrahim A. (2005). Novel perspectives on mucormycosis: pathophysiology, presentation, and management. CMR.

[98] Stappers M.H.T., Clark A.E., Aimanianda V., Bidula S., Reid D.M., Asamaphan P., Hardison S.E., Dambuza I.M., Valsecchi I., Kerscher B. (2018). Recognition of DHN-melanin by a C-type lectin receptor is required for immunity to Aspergillus. Nature.

[99] Stevenhagen A, and Furth R van. Interferon-gamma activates the oxidative killing of Candida albicans by human granulocytes. Clin. Exp. Immunol. (1993) 91, 170–175. 10.1111/j.1365-2249.1993.tb03374.x PMC1554646 8419079

[100] Templeton S.P., Rivera A., Hube B., Jacobsen I.D. (2018). Editorial: immunity to human fungal pathogens: mechanisms of host recognition, protection, pathology, and fungal interference. Front Immunol.

[101] ter Horst R., Jaeger M., Smeekens S.P., Oosting M., Swertz M.A., Li Y., Kumar V., Diavatopoulos D.A., Jansen A.F.M., Lemmers H. (2016). Host and environmental factors influencing individual human cytokine responses. Cell.

[102] Tischler BY, Tosini NL, Cramer RA, and Hohl TM. Platelets are critical for survival and tissue integrity during murine pulmonary Aspergillus fumigatus infection. PLOS Pathog. (2020) 16, e1008544. 10.1371/journal.ppat.1008544 PMC7252636 32407390

[103] Williams T.J., Harvey S., Armstrong-James D. (2020). Immunotherapeutic approaches for fungal infections. Curr Opin Microbiol.

[104] Torri A., Beretta O., Ranghetti A., Granucci F., Ricciardi-Castagnoli P., Foti M. (2010). Gene expression profiles identify inflammatory signatures in dendritic cells. PLoS One.

[105] Tucey T.M., Verma J., Harrison P.F., Snelgrove S.L., Lo T.L., Scherer A.K., Barugahare A.A., Powell D.R., Wheeler R.T., Hickey M.J. (2018). Glucose homeostasis is important for immune cell viability during candida challenge and host survival of systemic fungal infection. Cell Metab.

[106] van de Veerdonk F.L., Kullberg B.-J., Netea M.G. (2010). Pathogenesis of invasive candidiasis. Curr Opin Crit Care.

[107] van de Veerdonk F.L., Gresnigt M.S., Romani L., Netea M.G., Latgé J.-P. (2017). Aspergillus fumigatus morphology and dynamic host interactions. Nat Rev Microbiol.

[108] Vos T., Flaxman A.D., Naghavi M., Lozano R., Michaud C., Ezzati M., Shibuya K., Salomon J.A., Abdalla S., Aboyans V. (2012). Years lived with disability (YLDs) for 1160 sequelae of 289 diseases and injuries 1990–2010: a systematic analysis for the Global Burden of Disease Study 2010. Lancet.

[109] Walpole G.F.W., Grinstein S., Westman J. (2018). The role of lipids in host-pathogen interactions. IUBMB Life.

[110] Westman J, Moran G, Mogavero S, Hube B, and Grinstein S. Candida albicans Hyphal Expansion Causes Phagosomal Membrane Damage and Luminal Alkalinization. MBio (2018) 9. 10.1128/mBio.01226-18 PMC6134096 30206168

[111] Wisplinghoff H., Ebbers J., Geurtz L., Stefanik D., Major Y., Edmond M.B., Wenzel R.P., Seifert H. (2014). Nosocomial bloodstream infections due to Candida spp. in the USA: species distribution, clinical features and antifungal susceptibilities. Int J Antimicrob Agents.

[112] Wu C., Khan S.A., Lange A.J. (2005). Regulation of glycolysis—role of insulin. Exp Gerontol.

[113] Wu JF, Chen CH, Ni YH, Lin YT, Chen HL, Hsu HY, and Chang MH. Toll-like receptor and hepatitis B virus clearance in chronic infected patients: a long-term prospective cohort study in Taiwan. J. Infect. Dis. (2012) 206, 662–668. 10.1093/infdis/jis420 22740716

[114] Yeo J.C., Wall A.A., Luo L., Stow J.L. (2016). Sequential recruitment of Rab GTPases during early stages of phagocytosis. Cell Logist.

[115] Yoshida H., Matsui T., Yamamoto A., Okada T., Mori K. (2001). XBP1 mRNA is induced by ATF6 and spliced by IRE1 in response to ER stress to produce a highly active transcription factor. Cell.

[116] Young S.K., Shao Y.u., Bidwell J.P., Wek R.C. (2016). Nuclear matrix protein 4 is a novel regulator of ribosome biogenesis and controls the unfolded protein response via repression of gadd34 expression. J Biol Chem.

[117] Zhai B., Ola M., Rolling T., Tosini N.L., Joshowitz S., Littmann E.R., Amoretti L.A., Fontana E., Wright R.J., Miranda E. (2020). High-resolution mycobiota analysis reveals dynamic intestinal translocation preceding invasive candidiasis. Nat Med.

[118] Zhao S., Xi D., Cai J., Chen W., Xiang J., Peng N.a., Wang J., Jiang Y., Mei Z., Liu J. (2020). Rab20 is critical for bacterial lipoprotein tolerization-enhanced bactericidal activity in macrophages during bacterial infection. Sci China Life Sci.

[119] Zheng Z, Huang D, Wang J, Zhao K, Zhou Y, Guo Z, Zhai S, Xu H, Cui H, Yao H, et al.. QTLbase: an integrative resource for quantitative trait loci across multiple human molecular phenotypes. Nucleic Acids Res. (2020) 48, D983–D991. 10.1093/nar/gkz888 PMC6943073 31598699

