# Supplementary material for: Comparative host transcriptome in response to pathogenic fungi identifies common and species-specific transcriptional antifungal host response pathways
Source: Comput Struct Biotechnol J. 2020 Dec 26;19:647–63. doi: 10.1016/j.csbj.2020.12.036 (PMC7817431; doi:10.1016/j.csbj.2020.12.036)
Supplement: Supplementary data 1 [file mmc1.pdf]

Supplementary Figure 1

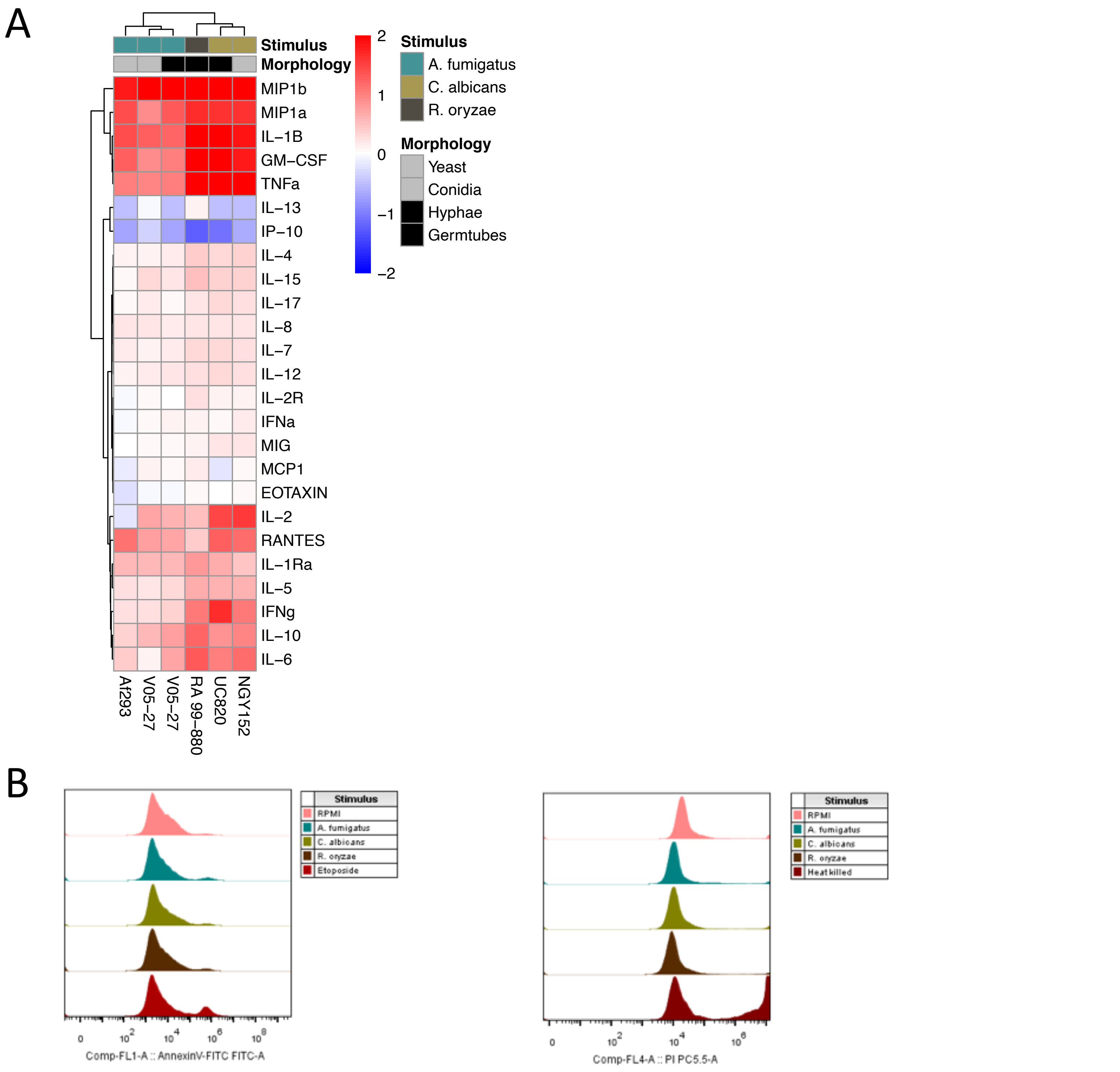

Supplementary Figure 1 (Figure S1). Functional validation of the fungal core host response and PBMCs viability upon 24 hour stimulation with *A. fumigatus*, *C. albicans* and *R. oryzae*; Related to Figure 4. (**A**) Heatmaps of cytokine production of PBMCs (n=9) stimulated with additional morphotypes and/or strains of *A. fumigatus*, *C. albicans* and *R. oryzae* compared to the unstimulated cells (RPMI). Means of the log10 fold-changes were calculated, and the data were clustered using hierarchical clustering on both axes (Euclidean distance, average linkage). Shade of red indicate upregulation, while shade of blue downregulation (see legend). (**B**) Viability of PBMCs (n=4) assessed by Annexin-V and PI staining after 24 hours stimulation with RPMI, *A. fumigatus*, *C. albicans* and *R. oryzae*, as well as positive controls for apoptosis (Etoposide, 50uM) and necrosis (heat-killing of cells for 1 minute at 65°C). The percentage of apoptotic cells gated on the singlet, $CD45^+$ cells are visualized by Annexin-V staining (left histogram), while the percentage of necrotic cells gated on the singlet, $CD45^+$ cells are visualized by PI staining (right histogram).
