# Supplementary material for: Comparative host transcriptome in response to pathogenic fungi identifies common and species-specific transcriptional antifungal host response pathways
Source: Comput Struct Biotechnol J. 2020 Dec 26;19:647–63. doi: 10.1016/j.csbj.2020.12.036 (PMC7817431; doi:10.1016/j.csbj.2020.12.036)
Supplement: Supplementary data 3 [file mmc3.docx]

| **Gene** | **Forward primer** | **Reverse primer** |
| --- | --- | --- |
| *TLR1* | CAGGCCCTCTTCCTCGTTAGA | TTCCTAAAGGTAGAAGCTGTTCTTCA |
| *TLR2* | GAATCCTCCAATCAGGCTTCTCT | GCCCTGAGGGAATGGAGTTTA |
| *TLR3* | TTTGCGAAGAGGAATGTTTAAATCT | CACCTATCCGTTCTTTCTGAACTG |
| *TLR4* | GGCATGCCTGTGCTGAGTT | CTGCTACAACAGATACTACAAGCACACT |
| *TLR5* | TGCTAGGACAACGAGGATCATG | GTTGCAGAAACGATAAAAGGCTATT |
| *TLR6* | CACAGAACAGCATTCCCAACA | AAAGAGCCCACGTTTGCTTTT |
| *TLR7* | GATTGAAACCTGACCAATTTGCT | AAATACGACATCGCCAATCTAAGG |
| *TLR8* | GGTCCTCTGCTCAGGGTGTCT | TGAATCCAGAAAACAACCACATG |
| *TLR9* | GGCCCCCGGCTTCTT | CAGGAGTGGTCCACTGTCTTGA |
| *TLR10* | GCATTCCCACCAGGTATCATAAAC | AAAGCCCACATTTACGCCTATC |
| *HK1* | CTGCTGGTGAAAATCCGTAGTGG | GTCCAAGAAGTCAGAGATGCAGG |
| *HK2* | TTGACCAGGAGATTGACATGGG | CAACCGCATCAGGACCTCA |
| *BiP (GRP78/HSPA5)* | GAAAGAAGGTTACCCATGCAGT | CAGGCCATAAGCAATAGCAGC |
| *ACTB* | AGAGCTACGAGCTGCCTGAC | AAGGGACTTCCTGTAACAACG |

**Table S2: List of primers used in the study;** related to figure 5 and 6.
